# Dual‐Criterion Approach Incorporating Historical Information to Seek Accelerated Approval With Application in Time‐to‐Event Group Sequential Trials

**DOI:** 10.1002/sim.70361

**Published:** 2026-01-23

**Authors:** Marco Ratta, Gaëlle Saint‐Hilary, Valentine Barboux, Mauro Gasparini, Donia Skanji, Pavel Mozgunov

**Affiliations:** ^1^ Department of Mathematical Sciences Polytechnic University of Turin Turin Italy; ^2^ Department of Statistical Methodology Saryga Tournus France; ^3^ Servier National Research Institute (IRIS) Servier Gif‐sur‐Yvette France; ^4^ MRC Biostatistics Unit Cambridge University Cambridge UK

**Keywords:** accelerated approval, group sequential design, historical data, probability of success, surrogate endpoint

## Abstract

The urgency of delivering novel, effective treatments against life‐threatening diseases has brought various health authorities to allow for Accelerated Approvals (AAs). AA is the “fast track” program where promising treatments are evaluated based on surrogate (short term) endpoints likely to predict clinical benefit. This allows treatments to get an early approval, subject to providing further evidence of efficacy, for example, on the primary (long term) endpoint. Despite this procedure being quite consolidated, a number of conditionally approved treatments do not obtain full approval (FA), mainly due to lack of correlation between surrogate and primary endpoint. This implies a need to improve the criteria for controlling the risk of AAs for noneffective treatments, while maximizing the chance of AAs for effective ones. We first propose a novel adaptive group sequential design that includes an early dual‐criterion “Accelerated Approval” interim analysis, where efficacy on a surrogate endpoint is tested jointly with a predictive metric based on the primary endpoint. Secondarily, we explore how the predictive criterion may be strengthened by historical information borrowing, in particular using: (i) historical control data on the primary endpoint, and (ii) the estimated historical relationship between the surrogate and the primary endpoints. We propose various metrics to characterize the risk of correct and incorrect early AAs and demonstrate how the proposed design allows explicit control of these risks, with particular attention to the family‐wise error rate (FWER). The methodology is then evaluated through a simulation study motivated by a Phase‐III trial in metastatic colorectal cancer (mCRC).

## 1 Introduction

Developing a new drug—from early phases to commercialization—is an extensive journey that requires substantial economic resources and time. This lengthy process is essential for rigorously evaluating many clinical aspects to guarantee the safety and efficacy of experimental treatments. However, accelerating drug developments becomes imperative for drugs filling unmet medical needs, where saving time may seriously impact the population survival [1].

To address this issue, Group Sequential Designs [2, 3, 4] have been widely used in the last decades, allowing for decision making at pre‐specified milestones during the study. There are many benefits of including interim analyses in a clinical trial: they permit the early termination of a trial, potentially reducing the number of patients exposed to an ineffective drug, or shortening the study duration in case of overwhelming efficacy. This provides the opportunity for patients with medical needs to receive an effective treatment earlier.

Interim analyses may have a sensible impact in reducing the total number of randomized patients and the study duration. However, due to the long time duration from randomization to endpoint observation in specific contexts such as in oncology, the number of events collected on the endpoint of interest at such an early time point is often not sufficiently large to make an informed decision on stopping or continuing the study. As a consequence, there is a growing interest in *surrogate endpoints*, defined as *biomarkers, laboratory measurements, radiographic images, physical signs or other measures allowing to predict clinical benefit* [5, 6]. These surrogate endpoints are linked to the primary endpoint of interest but can be observed in a shorter time frame.

The latter consideration, along with the urgency of delivering a prompt solution to life threatening diseases, led the US Food and Drug Administration (FDA) to institute the *Accelerated Approval* regulations in 1992 [5], a special program to give an early approval based on a *surrogate endpoint*. Under the current regulatory framework, AA is typically granted when substantial evidence of efficacy is demonstrated on a surrogate endpoint that is “reasonably likely to predict” clinical benefit, such as objective response rate or progression‐free survival (PFS) in oncology trials [5, 7]. In this framework, confirmatory trials are then required post‐approval to verify actual clinical benefit on the primary endpoint, commonly overall survival (OS). Despite the use of surrogate endpoints having many practical advantages, their consistency with the primary endpoint of interest still needs to be supported by actual data; as a result, once the AA is obtained—based on scientific relevance supporting the treatment efficacy on the surrogate endpoint—the company is required to provide valuable evidence of clinical benefit on the primary endpoint (under penalty of withdrawal of the product).

In a recent draft guidance by the FDA titled *Clinical Trial Considerations to Support Accelerated Approval of Oncology Therapeutics—Guidance for Industry* [7] (2023), two ways are detailed to conduct a clinical trial supporting an application for AA: (i) a *two‐trial approach* where one trial is conducted using a surrogate endpoint to support AA and a second confirmatory trial is conducted to verify clinical benefit on the long‐term primary endpoint, and (ii) a *one‐trial approach* where a single randomized controlled trial is conducted both to support AA and confirm clinical benefit. In particular, referring to the latter approach, two important points are that “the protocol should specify a plan to strongly control the overall false positive rate (type‐I error) for the endpoint supporting AA and the endpoint supporting verification of clinical benefit” and “the trial sample size should be chosen so that it has adequate power to detect a clinically meaningful and statistically significant improvement in *both* the endpoints for AA and verification of clinical benefit”.

Using a surrogate endpoint as a key endpoint for AA may seem a natural choice due to its wider and ready availability, however the validity of the surrogacy assumption and its quantification might not be easy to assess, potentially leading to incorrect decision‐making when surrogacy is incorrectly assumed. A review of the main methods for testing surrogacy is presented in [8]. In oncology, PFS has been demonstrated to be a valid surrogate for OS in many different cancer settings [9]. In particular, the surrogacy of PFS for OS in Metastatic Colorectal cancer (mCRC) has been demonstrated using different methodologies, for example, using Bayesian meta‐analytic regression [10] or via estimation of the correlation parameters [11].

Even if AA regulation has been vastly used since the program initiation, resulting in the AA of 192 drugs [12] (66 of which ongoing) as of October 2023, still 26 out of the 122 treatments that underwent confirmatory analysis failed to meet post‐marketing requirements, leading to a withdrawal of their approval by the FDA [13, 14]. Aside from safety reasons, this is mainly due to the lack of correlation between surrogate and primary endpoints. This shows that improving the current practice for assessing the AA criterion—based solely on testing a surrogate endpoint—is desirable. It may be beneficial not only from a regulatory and patients' perspective, by preventing noneffective treatments from entering the market erroneously, but also from a sponsor perspective, by increasing the chance that an AA is confirmed into a FA.

In this context, the current regulatory paradigm relies exclusively on hypothesis testing conducted on a surrogate endpoint, even though partial data on the primary endpoint are often available at the same time. Formal statistical testing on such incomplete primary endpoint data is generally not acceptable; however, these data could provide valuable predictive evidence supporting the likelihood of ultimate study success.

The main contribution of this work is the introduction of a novel *dual‐criterion approach* for AA within the one‐trial framework. The first criterion follows the conventional hypothesis testing on the surrogate endpoint, consistent with current regulatory practice. The second criterion, which constitutes the main innovation, introduces a predictive component based on the *Predictive Probability of Success* (PPoS) [15, 16, 17], that uses interim data from the primary endpoint to quantify the probability that the study will ultimately meet its confirmatory objective. The concept of PPoS has been used in different contexts, for instance in constructing futility stopping rules [18] or in predicting success of phase III studies based on phase II data [19]; however, its application in the context of AA has not been investigated previously.

However, estimating the PPoS can be particularly challenging when only a few primary endpoint observations are available, as limited data may lead to unstable or imprecise predictions. In this sense, leveraging historical or external information can help mitigate this limitation by providing additional context and improving the reliability of interim estimates. A secondary contribution of this work concerns the use of *historical borrowing* to inform the computation of the PPoS. Specifically, building upon existing borrowing methodologies, we explore how the incorporation of historical information from *multiple sources* through a Bayesian Dynamic Borrowing strategy using a *Robust Mixture Prior* [20, 21] can be applied in this context. This approach enables partial information sharing from relevant historical controls while maintaining robustness against prior‐data conflict, thereby improving the precision and stability of interim predictions.

The remainder of this article is organized as follows: Section 2 presents a detailed description of the methods. In Section 3, a motivating case study is introduced. Section 4 reports a simulation study comparing the single‐criterion and dual‐criterion approaches (DCA). In Section 5, the incorporation of historical information is discussed, and its added value within the dual‐criterion framework is investigated through a simulation study. Section 6 provides a sensitivity analysis, including the assessment of Bayesian metrics. Finally, Section 7 offers a discussion and outlines potential extensions of this work.

## 2 Methodology

### 2.1 Single‐Criterion One‐Trial Approach for Accelerated Approval (SCA)

Consider a randomized clinical trial, in which a new promising treatment is compared with a placebo or a standard of care using time‐to‐event endpoints. Suppose that a short(er) time endpoint—here, Progression Free Survival (PFS)—and a long(er) time endpoint—here, OS—are monitored along the trial, and consider PFS as a surrogate endpoint for OS, which is the primary endpoint of interest. A Group Sequential Design (GSD) [2, 3, 4] is employed, where a certain number of analyses in the set ℐ={1,…,I+1} (including I interim ones, and final one I+1)—are planned at pre‐specified information fractions on the primary endpoint. Moreover, let us suppose that, among the I interim analyses, some of them in the set ${ℐ}_{\text{AA}}\subset ℐ$ can lead to an AA request, and some of them in the set ${ℐ}_{\text{FA}}\subseteq ℐ$ can lead to FA request.

Let $r_{i,j}^{k}$ and $E_{i,j}^{k}$ be number of events occurred and the total exposure times at the *i*‐th stage of the trial (i.e., i=1 for the first interim look), for the *j*‐th endpoint (j∈{PFS, OS}) in the *k*‐th arm (k∈{C, T}, where C and T stands for *control* and *treatment* respectively); and let us define $\Delta_{i,j}=\left\{r_{i,j}^{k},E_{i,j}^{k},\;k=C,T\right\}$ as the generic set of data available on the j‐th endpoint at the *i*‐th interim analysis. Note that the total exposure time is defined as the sum of the individual exposure times across all patients, and each individual exposure time corresponds to the duration from randomization to either the occurrence of the endpoint or the end of the study, whichever occurs first.

Suppose the two time‐to‐event endpoints are exponentially distributed for both the control and treatment arms, and assume that the proportional hazards assumption holds. Let γ and θ be the hazard ratios respectively on the surrogate (PFS) and on the primary (OS) endpoints, and let $\lambda_{\text{PFS}}^{C}$ and $\lambda_{\text{OS}}^{C}$ be the control hazards on the surrogate and on the primary endpoints respectively. The number of events on OS, conditional on the model parameters, has a Poisson distribution

(1) $$\begin{array}{ll} r_{i,\text{OS}}^{C}|\lambda_{\text{OS}}^{C} & ∼\text{Poisson}\left(\lambda_{\text{OS}}^{C}E_{i,\text{OS}}^{C}\right)\\ r_{i,\text{OS}}^{T}|\theta ,\lambda_{\text{OS}}^{C} & ∼\text{Poisson}\left(\theta \lambda_{\text{OS}}^{C}E_{i,\text{OS}}^{T}\right) \end{array}$$

and similarly the number of PFS events at interim analyses

(2) $$\begin{array}{ll} r_{i,\text{PFS}}^{C}|\lambda_{\text{PFS}}^{C} & ∼\text{Poisson}\left(\lambda_{\text{PFS}}^{C}E_{i,\text{PFS}}^{C}\right)\\ r_{i,\text{PFS}}^{T}|\gamma ,\lambda_{\text{PFS}}^{C} & ∼\text{Poisson}\left(\gamma \lambda_{\text{PFS}}^{C}E_{i,\text{PFS}}^{T}\right). \end{array}$$

In a standard one‐trial approach, the study could be designed so that FA is requested if efficacy is achieved on the primary endpoint either at any of the interim analysis in the set ${ℐ}_{\text{FA}}$ (including the final analysis I+1), while AA is requested if clinical benefit is achieved on the surrogate endpoint at any interim analysis in the set ${ℐ}_{\text{AA}}$.

Let us define the “double null hypothesis” as the configuration where there is no treatment effect either on the surrogate nor on the primary endpoint (e.g., θ=γ=1), while we define an “alternative hypothesis” as the configuration where $\theta =\theta^{\#}<1$ and $\gamma =\gamma^{\#}<1$ (where $\theta^{\#}$ and $\gamma^{\#}$) represent the target hazard ratios on the primary and surrogate endpoints, respectively. Let us define, moreover “partial null scenarios” as the configurations where θ=1 and γ<1, meaning that the treatment is not effective on the primary endpoint but has some effect on the surrogate. Let $\pi_{\theta }^{0}(\cdot )$, $\pi_{\gamma }^{0}(\cdot )$, $\pi_{\lambda_{\text{OS}}^{C}}^{0}(\cdot )$ and $\pi_{\lambda_{\text{PFS}}^{C}}^{0}(\cdot )$ be the prior densities for the model parameters, properly chosen in order to reflect prior available information (including the use of vague priors when no prior information is available).

In a Bayesian framework, the success criterion for requesting a FA at the *i*‐th interim analysis is defined as

(3) $$\mathbb{P}\left(\theta <1\;|\;\Delta_{i,\text{OS}}\;;\;\pi_{\lambda_{\text{OS}}^{C}}^{0},\pi_{\theta }^{0}\right)>\eta_{i,\;\text{eff}}^{\text{OS}}$$

and the similarly success criterion for requesting an AA at the *i*‐th analysis is defined as

(4) $$\mathbb{P}\left(\gamma <1\;|\;\Delta_{i,\text{PFS}}\;;\;\pi_{\lambda_{\text{PFS}}^{C}}^{0},\pi_{\gamma }^{0}\right)>\eta_{i,\;\text{eff}}^{\text{PFS}}$$

where $\eta_{i,\;\text{eff}}^{\text{OS}}$ and $\eta_{\text{i, eff}}^{\text{PFS}}$ are the probability thresholds to claim efficacy, respectively, on the primary endpoint and on the surrogate endpoint at the *i*‐th stage of the trial. Early stops for futility at the *i*‐th stage of the trial may be also possible if $\mathbb{P}\left(\theta <1\;|\;\Delta_{i,\text{OS}}\;;\;\pi_{\lambda_{\text{OS}}^{C}}^{0},\pi_{\theta }^{0}\right)<\eta_{i,\;\text{fut}}^{\text{OS}}$. All the probabilities are computed with respect to the posterior distributions for θ and γ, which corresponding posterior densities are denoted by $g_{\theta }(\cdot |\Delta_{i,\text{OS}},\pi_{\lambda_{\text{OS}}^{C}}^{0},\pi_{\theta }^{0})$, and $g_{\gamma }(\cdot |\Delta_{i,\text{PFS}},\pi_{\lambda_{\text{PFS}}^{C}}^{0},\pi_{\gamma }^{0})$.

According to the recommendations in [7], the above‐mentioned probability thresholds should be calibrated in order to control the overall type I error under the double null scenario.

### 2.2 Dual‐Criterion One‐Trial Approach for Accelerated Approval (DCA)

In the previous section, a single‐criterion one‐trial approach for AA was based on PFS data collected at the time of the interim analysis. However, a number of events on OS are likely to be available at these times and may be employed in order to generate more convincing evidence that the experimental treatment has a positive benefit‐risk (which is not only based on statistical but also clinical aspects). Let us assume that some evidence on OS is available at the time of the interim analysis i (among the ones targeted for AA request), with its posterior density function $g_{\theta }(\cdot |\Delta_{i,\text{OS}},\pi_{\lambda_{\text{OS}}^{C}}^{0},\pi_{\theta }^{0})$, but that no enough evidence is available for an early decision regarding a FA request.

The Predictive Probability of Success (PPoS) of the current trial at the *i*‐th interim analysis is defined as the probability that the study demonstrates efficacy on OS at any future analysis (among the ones targeted for FA), conditional on the partial information collected at the *i*‐th interim analysis, which is

(5) $${\text{PPoS}}_{i}=\underset{\overset{h\in {ℐ}_{\text{FA}}}{h\geq i+1}}{\sum }{\text{PPoS}}_{i,h}$$

where ${\text{PPoS}}_{i,h}$ denotes the predictive probability computed at the *i*‐th interim analysis that the trial is successful at the *h*‐th interim look and not before, which is

(6) PPoSi,h=∫0+∞∫Ωm1ℙθ<1|Δ˜h,OS;πλOSC0,πθ0>ηh,effOSAND⋂k∈ℐFAi+1≤k≤h−1ℙθ<1|Δ˜k,OS;πλOSC0,πθ0<ηk,effOSfΔihΔ˜i+1,OS,…,Δ˜h,OS|Δi,OS,θ=tgθt|Δi,OS,πλOSC0,πθ0dΔihdt

The notation ${\tilde{\Delta }}_{*,\text{OS}}=\left\{{\tilde{r}}_{*,\text{OS}}^{C},{\tilde{r}}_{*,\text{OS}}^{T},{\tilde{E}}_{*,\text{OS}}^{C},{\tilde{E}}_{*,\text{OS}}^{T}\right\}$ refers to the predictive data at *‐th stage in the domain $\Omega ={\mathbb{N}}^{2}\times {\mathbb{R}}^{2}$, and $f_{\Delta_{ih}}$ represents the multivariate predictive data distribution at all the future stages of the study between the *i*‐th (excluded) and the *h*‐th (included) in the domain $\Omega^{m}=\Omega \times \Omega \times \cdots$ (m times, where m is the number of analyses targeted for FA request between the *i*‐th and the *h*‐th analysis). The probability is computed with respect to the posterior distribution for the treatment effect parameter on the primary endpoint $g_{\theta }(\cdot |\Delta_{i,\text{OS}},\pi_{\lambda_{\text{OS}}^{C}}^{0},\pi_{\theta }^{0})$ at the *i*‐th look.

A modification of the single criterion for AA at the *i*‐th interim look is proposed here by supplementing the condition on the surrogate endpoint in Equation (4) with a predictive criterion on the primary endpoint

(7) $${\text{PPoS}}_{i}>\eta_{i}^{\text{PPoS}}$$

where $\eta_{i}^{\text{PPoS}}$ may be chosen depending on the desired degree of confidence needed in the prediction. In the following sections, the two conditions in Equations (4) and (7) will be referred to as *PFS criterion* and *PPoS criterion* respectively. Note that, unlike the single‐criterion approach (SCA), where the analysis for the AA request was based solely on meeting the PFS criterion specified in Equation 4, the proposed DCA requires that both criteria in Equations (4) and (7) are simultaneously satisfied in order for the sponsor to request AA. This requirement is more stringent, but it also provides greater assurance on the efficacy of the experimental drug, hence greater confidence in a FA.

**FIGURE 1:**
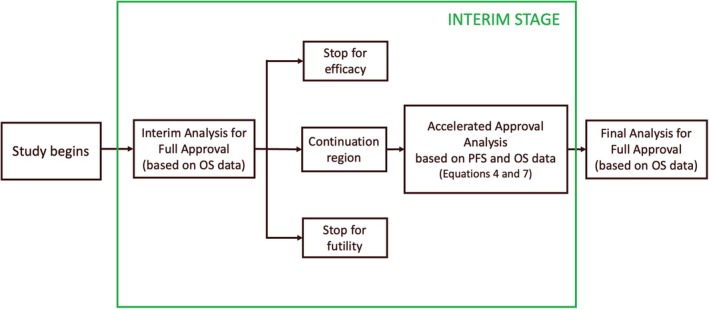
Illustration of the main analyses within the proposed trial design. One interim analysis—assessing both efficacy and futility—is presented here for FA based only on evidence on OS (following standard GSD rules); if no decision to stop is made based on interim data, an AA analysis is performed based on the DCA (Equation 4 and 7) using available evidence on both surrogate and primary endpoints. Only if the dual criterion is satisfied, the AA is requested. Regardless of the outcome of the AA analysis, if the study is not stopped for efficacy or futility, it continues until the Final Analysis, where a decision is made to request FA based on OS data only.

A schematic representation of the primary analyses underlying the proposed design (here illustrated in the simplified setting of a single interim analysis) is provided in Figure 1. At the time of the interim analysis, once a pre‐specified number of primary endpoint events has accrued, the data on the primary endpoint are analyzed according to the standard procedures employed in group sequential designs (GSD). A first decision is then made regarding whether to stop the trial for futility or to seek FA, based on the Bayesian hypothesis test for the primary endpoint effect θ. If a definitive conclusion cannot be drawn, for example if the interim data do not provide sufficient evidence to either reject or retain the null hypothesis, an AA assessment is subsequently performed by integrating the interim information from both the surrogate and primary endpoints. This assessment follows the dual‐criterion specified in Equations (4) and (7), and AA is pursued only when both conditions (i.e., statistical significance of the surrogate endpoint effect and a PPoS exceeding the pre‐specified threshold) are *simultaneously* met. Regardless of the interim outcome, the study continues to the final analysis, at which point the decision on whether to request FA is again determined solely on the basis of the updated primary endpoint data, using standard hypothesis testing in accordance with the GSD framework.

### 2.3 Control of Error Rates

In the design proposed in Section 3.1, an approval may be requested for the treatment either at one of the interim analysis times (FA or AA) or at the final analysis time (FA only). Multiple hypothesis testing due to the interim analyses and the two criteria for AA may lead to type I error inflation; hence, multiplicity adjustments are needed in order to control the family‐wise error rate (FWER).

In this context, it is important to distinguish between two types of error: *(i)* the risk of incorrectly requesting AA, and *(ii)* the risk of incorrectly requesting FA. Recall that the latter corresponds to a conventional hypothesis test on the primary endpoint. In contrast, the decision to request AA is based on the joint fulfillment of two criteria, one of which involves hypothesis testing on the surrogate endpoint. Therefore, rejecting the null hypothesis on the surrogate endpoint (γ=1) when it is in fact true is not, in isolation, to be considered an error, since it does not automatically lead to an AA request unless the second criterion is also satisfied.

We define FWER (also referred to as “Global Type I error” from now on) in our setting as the probability to be positive in at least one between FA and AA analysis at any stage of the trial (interim or final) when there is no treatment effect on the primary endpoint (θ=1). This quantity should be interpreted as the overall risk of incorrect decision‐making, either through an incorrect application for AA or an incorrect request for FA. However, since a global type I error can arise through multiple pathways, it is useful to distinguish among the specific sources of error that contribute to this overall risk.

Let us define the following quantities:

- *FA rate (denoted as* $\alpha_{\theta }^{\text{FA}}$): the probability to reject the null hypothesis on the primary endpoint (either at any of the interim or at the final analysis), regardless of whether AA is requested or not at any previous stage. Note that this quantity does not depend on the treatment effect on the surrogate endpoint (see Equation 3). Notice that when there is no effect on the primary endpoint (θ=1), this probability represents the risk of incorrectly requesting FA.
- *Accelerated Approval rate (denoted as* $\alpha_{\gamma ,\theta }^{\text{AA}}$): the probability to fulfill the two criteria for FA analysis based on the DCA (Equations 4 and 7); it depends on both the true treatment effect on the surrogate endpoint γ and the true treatment effect on the primary endpoint θ. Notice that when there is no effect on the primary endpoint (θ=1), this probability represents the risk of incorrectly requesting an AA at any interim analysis.
- *Confirmed Accelerated Approval rate (denoted as* $\alpha_{\gamma ,\theta }^{\text{CAA}}$): the probability that the dual‐criterion for AA has been fulfilled (at any interim analysis), and that the criterion for FA (at any subsequent analysis) is also fulfilled; it depends on both the true treatment effect on the surrogate endpoint γ and the true treatment effect on the primary endpoint θ. Notice that when there is no effect on the primary endpoint (θ=1), this probability may be intended as the risk of incorrectly requesting an AA at interim, and FA at any subsequent analysis.
- *Global Approval rate (denoted as* $\alpha_{\gamma ,\theta }^{G}$): it is the probability to be positive in at least one among AA analysis and FA analysis at any of the interim analysis or at the final analysis; it depends on both the true treatment effect on the surrogate endpoint γ and the true treatment effect on the primary endpoint θ. Notice that when there is no effect on the primary endpoint (θ=1), this probability may be intended as the risk of incorrectly requesting at least one among AA and FA, which represents the global type I error rate.

Note that both AA and FA analysis contribute to the global Approval rate, then the following decomposition holds:

(8) $$\alpha_{\gamma ,\theta }^{G}=\alpha_{\gamma ,\theta }^{\text{AA}}+\alpha_{\theta }^{\text{FA}}-\alpha_{\gamma ,\theta }^{\text{CAA}}$$

Notice that the minus sign arises from the inclusion‐exclusion principle, which states that for any two events A and B, the probability that at least one occurs is given by P(A∪B)=P(A)+P(B)−P(A∩B). In our context, the *global type I error* is defined as the probability that a false positive conclusion is reached in at least one of the two analyses, AA or FA. As such, the probability of a type I error in either analysis is expressed as the sum of the type I error probabilities for the individual analyses (namely $\alpha_{\gamma ,\theta }^{\text{AA}}$ and $\alpha_{\theta }^{\text{FA}}$), minus the probability that both yield false positive results (namely $\alpha_{\gamma ,\theta }^{\text{CAA}}$). The subtraction term ensures that this last term $\alpha_{\gamma ,\theta }^{\text{CAA}}$ is not counted twice, as it is already included in both $\alpha_{\gamma ,\theta }^{\text{AA}}$ and $\alpha_{\theta }^{\text{FA}}$.

Since $\alpha_{\gamma ,\theta }^{\text{AA}}$ depends on the two criteria in Equations (4) and (7), then we also define

- *PFS Accelerated Approval rate (denoted as* $\alpha_{\gamma }^{\text{AA‐PFS}}$): the probability to meet PFS criterion for AA, that is, the probability to claim statistical significance in treatment effect on the surrogate endpoint, when the latter is equal to γ.
- *OS Accelerated Approval rate (denoted as* $\alpha_{\gamma ,\theta }^{\text{AA‐PPoS}}$): the probability to meet the PPoS criterion for AA, which is the probability to have a high PPoS on the primary endpoint at the interim analysis.

Note that the relationship between $\alpha_{\gamma ,\theta }^{\text{AA}}$, $\alpha_{\theta ,\gamma }^{\text{AA‐PPoS}}$ and $\alpha_{\gamma }^{\text{AA‐PFS}}$ depends on the patient level correlation between the surrogate and primary endpoints, in particular the following holds

(9) $$\alpha_{\gamma }^{\text{AA‐PFS}}\alpha_{\gamma ,\theta }^{\text{AA‐PPoS}}\leq \alpha_{\gamma ,\theta }^{\text{AA}}\leq \min\left(\alpha_{\gamma }^{\text{AA‐PFS}},\;\alpha_{\gamma ,\theta }^{\text{AA‐PPoS}}\right).$$

From Equation (8) and (9), it follows that

(10) $$\alpha_{\gamma ,\theta }^{G}<\alpha_{\gamma }^{\text{AA‐PFS}}+\alpha_{\theta }^{\text{FA}}$$

For requesting an AA, a standard requirement imposed by health authorities [7] is that Global type I error is maintained under the *double null scenario* θ=γ=1 [7] under a pre‐specified level $\omega^{G}$. This control depends directly on the choice of the probability thresholds $\eta_{i,\;\text{eff}}^{\text{PFS}}$ and $\eta_{i,\;\text{fut}}^{\text{PFS}}$ (which contribute to $\alpha_{\gamma =1}^{\text{AA‐PFS}}$), $\eta_{i,\text{eff}}^{\text{OS}}$ and $\eta_{i,\text{fut}}^{\text{OS}}$ (which contribute to $\alpha_{\theta =1}^{\text{FA}}$) and $\eta^{\text{PPoS}}$ (which contributes to $\alpha_{\gamma =1,\theta =1}^{\text{AA‐PPoS}}$). Many distinct combinations of the latter may be employed so that $\alpha_{\gamma =1,\theta =1}^{G}<\omega^{G}$.

In our context, exploiting the inequality in Equation (10) in order to control the global type I error rate under the double null scenario, we propose to split $\omega^{G}$ between $\alpha_{\gamma =1}^{\text{AA‐PFS}}$ and $\alpha_{\theta =1}^{\text{FA}}$ (so that $\alpha_{\gamma =1}^{\text{AA‐PFS}}+\alpha_{\gamma =1,\theta =1}^{\text{FA}}=\omega^{G}$), choosing accordingly the probability thresholds for PFS testing ($\eta_{i,\;\text{eff}}^{\text{PFS}}$, $\eta_{i,\;\text{fut}}^{\text{PFS}}$) and for OS testing ($\eta_{i,\text{eff}}^{\text{OS}}$ and $\eta_{i,\text{fut}}^{\text{OS}}$) based on any standard GSD rule for example, alpha‐spending functions [22]. Under this splitting strategy, no portion of the nominal level $\omega^{G}$ is allocated to the PPoS criterion; therefore, the same allocation can also be applied to the SCA. It is important to note that the fact that no portion of $\omega^{G}$ is allocated to the PPoS criterion does not imply that the criterion defined in Equation 7 is without impact. Rather, the PPoS criterion is applied in addition to the hypothesis test on the surrogate endpoint, with the goal of *reinforcing* the evidence in support of an AA request. This strategy also ensures control of the global type I error rate, even in the presence of potential misspecification of the PPoS model.

In principle, a control of the global type I error could also be achieved by splitting $\omega^{G}$ exploiting Equation (8). However, not including $\alpha_{\gamma =1,\theta =1}^{\text{AA‐PPoS}}$ in the split driven by Equation (10) has two main advantages: first it leads to type I error rates strictly below the nominal level (since it relies on a strict inequality), second it assures that the Global type I error is maintained non depending on predictions, but rather based solely on concurrent data. We acknowledge that this approach is conservative and does not fully utilize the nominal level $\omega^{G}$. However, the rationale behind this choice is that allocating a portion of the nominal level explicitly to the PPoS criterion may be difficult to justify to regulatory authorities, given the potential bias arising from the limited amount of primary endpoint data typically available at the time of the interim analysis.

As a consequence of this choice the control of the FWER under the double null scenario is guaranteed for any value of $\eta^{\text{PPoS}}$, which then remains to be set.

Instead, we propose to use the protection of the FWER under partial null scenarios as a rationale for the choice of $\eta^{\text{PPoS}}$. Let us generically define a “safeguard scenario” as any partial null scenario [θ=1, $\gamma =\gamma^{*}]$ where it is desirable a control of the false positive rate, then the PPoS threshold may be set as follows:

(11) $$\eta_{*}^{\text{PPoS}}=\underset{\eta^{\text{PPoS}}\in [0,1]}{\text{arg min}}\;\alpha_{\gamma =\gamma^{*},\theta =1}^{G}\;\;\;\;\;\text{s.t.}\;\;\;\;\;\alpha_{\gamma =\gamma^{*},\theta =1}^{G}<\omega^{\text{SG}}$$

where $\omega^{\text{SG}}$ is a pre‐determined level of control of the false positive rate under the “safeguard scenario.” Note that the further “safeguard scenario” is from the double null scenario (i.e., $\gamma^{*}<<1$), the higher $\eta^{\text{PPoS}}$ and vice‐versa. Additionally, for a given treatment effect on the surrogate endpoint, a lower value of $\eta^{\text{PPoS}}$ leads to a higher rate of AA requests, which is favorable if the treatment is actually effective, but implies an increase in incorrect AA rates if the treatment has no effect on the primary endpoint. On the contrary, high values of $\eta^{\text{PPoS}}$ would decrease the number of incorrect AA requests in case of noneffective treatments, but may limit the number of AA in case of effective ones.

We note that, within the framework considered in this work, an AA request, even if granted by the regulatory authority, does not guarantee eventual Full Approval (FA). As a result, the parameter $\eta^{\text{PPoS}}$ does not influence the type I error rate associated with FA. Nevertheless, high values of $\eta^{\text{PPoS}}$ are recommended in order to better align AA requests with FA request, thereby increasing the probability that an AA ultimately leads to FA.

### 2.4 Specification of Prior Distributions

In the single criterion one‐trial approach (SCA approach) proposed in Section 2.1, we first propose to use weak priors in the FA analysis for the hazard ratio θ and the control hazard $\lambda_{\text{OS}}^{C}$, as well as in the AA analysis for γ and $\lambda_{\text{PFS}}^{C}$. This ensures that decision‐making is approximately entirely driven by concurrent data, while achieving almost equivalence between Bayesian and frequentist analyses (e.g., based on the log‐rank test). Consistently with the specification mentioned in [23], the following prior distributions are used:

(12) $$\begin{array}{ll} \lambda_{\text{OS}}^{C} & ∼\text{Lognormal}\;\left(0,100\right)\;\;\;\;\;\;\lambda_{\text{PFS}}^{C}∼\text{Lognormal}\;\left(0,100\right)\\ \gamma & ∼\text{Lognormal}\;\left(0,4\right)\;\;\;\;\;\;\theta ∼\text{Lognormal}\;\left(0,4\right) \end{array}$$

The standard deviations in the prior distributions for γ and θ are set such that their *effective sample sizes* are equal to one. Conceptually, this implies that the Fisher information conveyed by each prior distribution is equivalent to that obtained from observing a single patient.

In the novel DCA detailed in Section 3.1, the PPoS criterion is introduced to strengthen the AA analysis by incorporating data on the primary endpoint. In this approach, for sake of first evaluation, the same prior distributions employed for the SCA approach in Formulas (12) are used for θ, γ, $\lambda_{\text{OS}}^{C}$ and $\lambda_{\text{PFS}}^{C}$. This choice may be sensible for example when no historical information is available for any of the model parameters, or if it is believed that the available prior information is significantly different from what is expected to be observed in the current trial. We acknowledge that this assumption is often not reflective of typical confirmatory settings, which are usually conducted when some prior information on the model parameters is already available. Nonetheless, the proposed approach remains a valid option in cases where the sponsor opts (or the regulator asks) not to incorporate such information at the *analysis* stage of the trial. This does not imply that existing knowledge should be disregarded entirely; rather, external data may still play an important role at the *design stage*, for example, as inputs for sample size calculation or other design‐related decisions. The use of historical information to inform the prior distribution employed in the computation of the PPoS is examined in Section 5.

## 3 Case Study

### 3.1 Motivating Example

In this section the proposed methodology will be applied in the context of a phase III trial in mCRC. Although all the data used for this example are fictive, the design assumptions made for this case study are inspired by a real study.

The primary endpoint of our case study is OS, defined as the time from randomization to death, and the secondary (surrogate) endpoint is Progression Free Survival (PFS), defined as the time from randomization to disease progression or death (whichever happens first). The hazard ratio (HR) is used as a measure of the treatment effect for both endpoints.

The trial compares the experimental treatment to a control using a 1:1 randomization. The global type I error $\alpha_{\gamma =1,\theta =1}^{G}$, that is, the overall probability to requesting a marketing approval for a noneffective treatment (either via Accelerated or FA) must be controlled at a level $\omega^{G}=2.5\%$ one‐sided, and an equally weighted Bonferroni split between $\alpha_{\gamma =1}^{\text{AA‐PFS}}$ and $\alpha_{\theta =1}^{\text{FA}}$ is chosen according to Equation (10). This implies that half the nominal level $\omega^{G}=2.5\%$ is assigned to the probability to apply for AA and FA.

Assuming a maximum of 500 patients can be enrolled in the study and an accrual rate of 30 patients per month, supposing a median OS of 8.5 months for the control arm and targeting a 29% reduction in OS on the treatment arm (θ=0.71, corresponding to 3.5 months increase in median OS from baseline), and a 1.25% FA type I error one‐sided, a total of 424 events is required to achieve 90% power. Computation of the sample size has been performed using the R [24] package ‘rpact’ [25].

One single interim analysis is planned—both to test treatment efficacy on the primary endpoint and to assess the AA criteria—after 84 (20%) OS events are observed. Assuming a median PFS of 2.1 months for the control arm and a 47.5% reduction in PFS (γ=0.525, corresponding to 1.9 months increase in median PFS from baseline), 170 PFS events are expected at the time of the interim analyses with a marginal power (probability to get a statistical significant surrogate treatment effect) of 97.5%.

An O'Brien‐Fleming spending function is chosen to set the probability thresholds for efficacy on the primary endpoint at the time of the interim analysis and at the time of the final analysis, which are respectively $\eta_{\text{I, eff}}^{\text{OS}}=0.9999$ and $\eta_{\text{I, eff}}^{\text{OS}}=0.9875$. No futility interim analyses are set for the sake of simplicity ($\eta_{\text{I, fut}}^{\text{OS}}=0$. According to the Bonferroni split of $\alpha_{\gamma =1,\theta =1}^{G}$ between the two endpoints PFS and OS, a threshold $\eta_{\text{I, eff}}^{\text{PFS}}=0.9875$ is set to keep $\alpha_{\gamma =1}^{\text{AA‐PFS}}$ below its nominal level 1.25% under γ=1 (and accordingly the FWER $\alpha_{\gamma =1,\theta =1}^{G}$ below $\omega^{G}=2.5\%$ under the double null scenario of γ = 1 and θ = 1). A threshold for the PPoS criterion $\eta_{*}^{\text{PPoS}}=0.91$ is moreover obtained from numerical simulations in order to control the global type I error rate $\alpha_{\gamma =\gamma^{*},\theta =1}^{G}$ at $\omega^{\text{SG}}=2.5\%$ level under the safeguard scenario of $\gamma^{*}$ = 0.525 and θ = 1 (corresponding to a situation where the treatment is not effective on the primary endpoint but it exhibits the target treatment effect on the surrogate endpoint)). The threshold is computed assuming that the study data for the control arm follow the design assumptions (i.e., median OS of 8.5 months).

### 3.2 Analysis

To illustrate the practical implementation of the proposed approach, we present an analysis based on a fictitious trial, for which the data have been generated numerically. Specifically, in this example, PFS and OS data are simulated under the assumption of no treatment effect on the primary endpoint (θ=1) and a moderate treatment effect on the surrogate endpoint (γ=0.6).

Assume that, for an ongoing phase III trial, the following data are available at the time of the interim analysis:

- For the surrogate endpoint: $r_{\text{1,PFS}}^{C}=104$, $E_{\text{1,PFS}}^{C}=283$, $r_{\text{1,PFS}}^{T}=88$, $E_{\text{1,PFS}}^{T}=356$;
- For the primary endpoint: $r_{\text{1,OS}}^{C}=48$, $E_{\text{1,OS}}^{C}=495$, $r_{\text{1,OS}}^{T}=36$, $E_{\text{1,OS}}^{T}=560$.

A summary of the results of the analysis is in Table 1.

**TABLE 1:** Summary of the case study analysis.

Interim analysis (IF = 0.2)						Final analysis	
ℙ(γ<1)	$\eta_{\text{I,eff}}^{\text{PFS}}$	ℙ(θ<1)	$\eta_{\text{I,eff}}^{\text{OS}}$	PPoS	$\eta^{\text{PPoS}}$	ℙ(θ<1)	$\eta_{\text{II,eff}}^{\text{OS}}$
0.998	0.9875	0.967	0.9999	0.793	0.9	0.88	0.9875

Testing the treatment efficacy on the primary endpoint at the interim analysis, we get $\mathbb{P}\left(\theta <1\;|\;\Delta_{\text{1,OS}}\;;\;\pi_{\lambda_{\text{OS}}^{C}}^{0},\pi_{\theta }^{0}\right)=0.967$, which is lower than the pre‐specified threshold $\eta_{\text{1,eff}}^{\text{OS}}=0.9999$. Since not enough evidence is provided to stop early for efficacy, we proceed with the AA analysis. Testing the treatment efficacy on the surrogate endpoint at the interim analysis, we get $\mathbb{P}\left(\gamma <1\;|\;\Delta_{\text{1,PFS}}\;;\;\pi_{\lambda_{\text{PFS}}^{C}}^{0},\pi_{\gamma }^{0}\right)=0.998$, which is greater than the pre‐specified threshold $\eta_{\text{1,eff}}^{\text{PFS}}=0.9875$. As a consequence, the PFS criterion is satisfied (the treatment seems effective in reducing the risk on PFS), and an AA request is recommended using the SCA.

Testing the PPoS criterion on the primary endpoint, we obtain a PPoS of 0.793. Although the PFS criterion is satisfied, the PPoS is not greater than the pre‐specified threshold of $\eta_{\text{PPoS}}^{\text{OS}}=0.91$, hence, there is not enough evidence to recommend an AA according to the DCA, and further data are needed to make a decision.

At the end of the trial, when 424 planned events on OS have been observed, let's assume that we observe on the primary endpoint $r_{\text{2,OS}}^{C}=215$, $E_{\text{2,OS}}^{C}=2600$, $r_{\text{2,OS}}^{T}=209$, $r_{\text{2,OS}}^{T}=2836$. Testing the treatment efficacy on the primary endpoint at the final analysis, we get $\mathbb{P}\left(\theta <1\;|\;\Delta_{\text{2,OS}}\;;\;\pi_{\lambda_{\text{OS}}^{C}}^{0},\pi_{\theta }^{0}\right)=0.88$, which is lower than the pre‐specified threshold $\eta_{\text{2,eff}}^{\text{OS}}=0.9875$, hence not enough evidence against the null hypothesis is provided and a FA cannot be requested.

This example shows the added value of our methodology: while relying on data on the surrogate endpoint only would have been misleading (bringing us to an incorrect AA request), reinforcing the AA request criteria with the PPoS criterion helped us in avoiding the wrong AA request for an ineffective treatment.

## 4 Numerical Evaluation

This section presents a simulation study designed to evaluate the approach introduced in Section 2 across a range of scenarios. The primary objective is to evaluate and compare the performance of the DCA relative to the SCA across various parameter settings, with particular emphasis on potential deviations from the design assumptions regarding the control parameter and the surrogate treatment effect parameter.

### 4.1 Setting

The design assumptions, as well as the probability thresholds used for decision making and the available historical information, are the same as in Section 3.1.

The performance of the SCA and the DCA, introduced in Section 2, is evaluated across 12 scenarios (Table 2). The model parameters $\lambda_{\text{OS}}^{C}$, γ, and θ are systematically varied to represent possible deviations from the design assumptions.

**TABLE 2:** Considered scenarios: for three different median OS on current control, 5 scenarios—2 for effective treatment (listed with the letter A) and 3 for noneffective treatments (listed with the letter N) are simulated varying γ, θ, and $\lambda_{C}^{\text{OS}}$.

	Scenarios											
	A0 LOW	A1 LOW	N0 LOW	N1 LOW	A0	A1	N0	N1	A0 HIGH	A1 HIGH	N0 HIGH	N1 HIGH
γ	0.39	0.75	1	0.525	0.39	0.75	1	0.525	0.39	0.75	1	0.525
θ	0.71	0.71	1	1	0.71	0.71	1	1	0.71	0.71	1	1
median(OS)	7	7	7	7	8.5	8.5	8.5	8.5	10	10	10	10

*Note*: Median OS for the control is retrieved by the formula $\text{median(OS)}=\log(2)/\lambda_{C}^{\text{OS}}$, which is valid for exponential OS.

Effective treatments (θ=0.71) are denoted by the letter “A” (standing for *alternative*), whereas noneffective treatments (θ=1) are denoted by the letter “N” (standing for *null*). Scenarios labeled with the number “0” represent situations of agreement between the design assumptions and the concurrent data in terms of treatment effects γ and θ, while scenarios labeled with other indices correspond to deviations of the concurrent data from the design assumptions.

For each main scenario, three sub‐scenarios are further analyzed, varying the control parameter. In particular, scenarios labeled “LOW” and “HIGH” correspond to inferior (median(OS) = 7 months) and superior (median(OS) = 10 months) concurrent controls, respectively, with respect to the design assumptions, whereas unlabeled scenarios indicate perfect agreement between the control parameter and the design assumptions. It is worth noting that scenario N1 represents the previously defined *safeguard scenario* used to calibrate the threshold for the PPoS criterion, $\eta^{\text{PPoS}}$.

For this analysis, data were generated assuming no patient‐level correlation between surrogate and primary endpoints; the same analysis made assuming a correlation of 0.45 is presented in the Supporting Information as a sensitivity analysis.

For each of the 12 scenarios, 1000 trials are simulated, and results are obtained making use of an approximation of the posterior distributions for the model parameters obtained via Markov Chain Monte Carlo (MCMC) obtain using the R [24] package RJags [26].

### 4.2 Evaluation Metrics

The two approaches under comparison, namely the SCA and the DCA, are evaluated according to the following performance metrics:

- Accelerated Approval Rate (AA), which is approximated as the fraction of the total simulated trials that is positive in the AA analysis.
$$\text{AA}=\frac{\text{\# Accelerated Approvals}}{\text{\# Trials Simulated}}$$
- Confirmation Rate (CR) which is approximated as the fraction of the simulated trials passing the FAl Analysis at the final analysis in Equation (3) among the ones which pass the AA Analysis.
$$\text{CR}=\frac{\text{\# (Accelerated Approval}\cap \text{Full Approval)}}{\text{\# Accelerated Approval}}$$
- Full Approval Rate (FA) which is defined as the fraction of the total simulated trial which is positive in the FA Analysis (either at the interim or at the final stage) in Equation (3).
$$\text{FA}=\frac{\text{\# Full Approval}}{\text{\# Trials Simulated}}$$
- Global type I error rate (G‐t1E) which is approximated—only for ineffective treatments (θ=1)—as the fraction of the total simulated trial which passes at least one among FA Analysis in Equation (3) or Accelerated Approval Analysis.
$$\text{G‐t1E}=\frac{\text{\# (Full Approval}\cup \text{Accelerated Approval)}}{\text{\# Trials Simulated}}$$

### 4.3 Results

Table 3 provides a comprehensive summary of the results. Each row corresponds to a specific scenario defined in Table 2. The first column, labeled *Scenario*, identifies the respective scenario, while the subsequent columns report the four performance metrics considered, namely, the *Accelerated Approval Rate*, *Confirmation Rate*, *Full Approval Rate*, and *Global Type I Error Rate*. For each metric, the results are presented under two methodological frameworks: the *Single‐Criterion Approach* (SCA) and the *Dual‐Criterion Approach* (DCA).

**TABLE 3:** Comparison between single‐criterion approach (SCA) and dual‐criterion approach (DCA).

Scenario	Accelerated approval rate		Confirmation rate		Full approval rate		Global type I error rate	
	SCA	DCA (no borrowing)	SCA	DCA (no borrowing)	SCA	DCA (no borrowing)	SCA	DCA (no borrowing)
A0 LOW	99.8	40.9	91.3	98.8	91.3	91.3	—	—
A1 LOW	39.5	15.8	91.6	98.1	91.3	91.3	—	—
N0 LOW	1.1	0.0	—	—	1.2	1.2	2.3	1.2
N1 LOW	96.8	1.6	—	—	1.2	1.2	96.9	2.6
A0	100	44.1	91.1	98.2	91.1	91.1	—	—
A1	42.8	19.4	91.8	97.9	91.1	91.1	—	—
N0	1.4	0.0	—	—	1.2	1.2	2.6	1.2
N1	97.6	1.5	—	—	1.1	1.1	97.7	2.3
A0 HIGH	100	45.6	90.8	98.2	90.8	90.8	—	—
A1 HIGH	47.3	21.8	91.1	97.7	90.8	90.8	—	—
N0 HIGH	1.0	0.0	—	—	1.2	1.2	2.2	1.2
N1 HIGH	99.1	1.8	—	—	1.2	1.2	99.1	2.5

In terms of AA, results show that under the SCA approach, the probability of passing the analysis for an AA request is consistently high whenever the surrogate endpoint shows meaningful treatment effects, reaching almost 100% in settings where a strong surrogate treatment effect is shown (A0 and N1 scenarios) and remaining between 39% and 47% under moderate effects (A1 scenarios). In null scenarios (N0), it closely aligns with the nominal level of $\omega^{G}/2=1.25\%$. When moving to the DCA, however, the inclusion of the PPoS criterion, based on partially observed primary endpoint data, substantially lowers the probability of meeting the conditions for an AA request in all configurations, often reducing it by half or more compared with the SCA (e.g., 100% vs. 45.6% in A0‐HIGH, 42.8% vs. 19.4% in A1). This reduction is a direct consequence of the additional evidentiary requirement introduced by the PPoS, which demands a consistent signal of efficacy on both the surrogate and the primary endpoint, even when the latter is only partially observed. As a result, the DCA acts as a more conservative filter at the interim stage, considerably limiting the number of trials that would proceed to an AA request.

This more stringent decision rule also affects the consistency between the interim and final analyses, as reflected by the Confirmation Rate (CR). While the SCA yields CR values around 91%, indicating that roughly one in ten trials that requested AA would eventually fail to meet the requirements for a FA request, the DCA increases the CR to approximately 98% across all scenarios. This improvement means that when a trial passes the interim analysis under the DCA, it is much more likely to ultimately meet the conditions for FA. In other words, the DCA substantially enhances the reliability of the process, ensuring that interim decisions based on the surrogate and predictive information are more coherent with the final primary endpoint results. As expected, the FA Rate itself remains virtually unchanged between the two methods, around the nominal level of 90% in alternative scenarios and around 1.25% under the null, since both rely on the same confirmatory analysis of the primary endpoint. What differentiates the two approaches, therefore, is not the ultimate probability of final approval, but the coherence between the intermediate and final phases: the SCA produces more early requests, with a higher chance of later rejection, whereas the DCA results in fewer, but more reliable, requests.

In terms of G‐t1E, under the SCA, the global type I error is around the nominal level of 2.5% under the double null scenarios (with minor deviations due to simulation error) but becomes severely inflated under the partial null configurations (N1), reaching values above 95%. This inflation arises from the inconsistency between the large treatment effect observed on the surrogate endpoint and the absence of a corresponding effect on the primary endpoint, which ultimately results in a high probability of erroneously proceeding with an AA request when the treatment provides no true benefit on the primary endpoint. In contrast, the DCA maintains tight control of the FWER across all scenarios, consistently keeping it close to the nominal level of 2.5% under the *safeguard scenarios* (N1‐LOW, N1, N1‐HIGH) and below the nominal level under the double null scenarios (N0‐LOW, N0, N0‐HIGH). This demonstrates the robustness of the dual‐criterion rule in preventing false requests for early approval and maintaining overall statistical integrity.

Notably, in both approaches, and in the presence of a surrogate treatment effect, an increase in the AA is observed for larger values of the median control OS (e.g., 40.9% in scenario A0‐LOW, 44.1% in scenario A0, and 45.6% in scenario A0‐HIGH). This can be attributed to the fact that higher survival in the control arm delays the timing of the interim analysis, which is consequently performed after a greater number of PFS events have accrued, ultimately yielding a more precise estimation of the surrogate treatment effect.

Overall, these results indicate that the DCA framework offers clear advantages in aligning the outcomes of the AA analysis with those of the FA analysis, thereby improving the coherence between AA requests and subsequent FA requests. By integrating partial information from the primary endpoint through the PPoS, the DCA limits the number of incorrect or premature requests while ensuring that those that do proceed are more likely to be confirmed at the end of the study. However, this greater reliability comes at the cost of a marked reduction in the probability of passing the AA analysis. The high uncertainty associated with the PPoS, due to the limited amount of primary endpoint data available at the interim stage, makes the DCA highly conservative in identifying trials suitable for early submission.

In the next section, we explore how incorporating historical data can mitigate this limitation by improving the precision of PPoS estimation, thereby enhancing the efficiency of the DCA while preserving its robustness.

## 5 Augmenting DCA via Historical Information Borrowing

In the new framework detailed in Section 3.1, the PPoS criterion is introduced to strengthen the AA analysis by incorporating data on the primary endpoint. However, the number of events on the primary endpoint at the time of the interim analysis is likely to be small, and this may lead to a poor estimation of the treatment effect (due to the high sampling variance), thus limiting the benefit of the PPoS criterion itself. To avoid this risk, different types of informative priors can be considered, aiming to improve the parameter estimation and to enhance the AA request.

In the following paragraphs, building upon existing historical borrowing methodologies described in the literature, we propose two distinct approaches for incorporating historical information, namely to borrow information on the control parameter $\lambda_{\text{OS}}^{C}$ and on the treatment effect parameter θ. This approach will inevitably make the decision process dependent not only on the current trial data but also on supportive studies; therefore, a careful assessment of the relevance and appropriateness of the historical information is essential, as well as an adequate handling of potential heterogeneity between current and external data.

### 5.1 Borrowing Historical Control Information

In this section, we adopt the same meta‐analytic predictive (MAP) framework proposed by Roychoudhury et al. [23], as it provides a coherent Bayesian approach for dynamically borrowing information from historical control data. This methodology is particularly relevant in our context, where leveraging external evidence can improve the estimation of control parameters and enhance the precision of predictive analyses. While Roychoudhury et al. describe a general piece‐wise exponential model for OS (OS), we employ a simplified version assuming a single exponential distribution for OS, which adequately represents the survival dynamics observed in our data while preserving the core structure of their approach.

Let us assume that data for the control arm are available, for example, from a literature review, on the primary endpoint in H historical trials. Suppose that we want to leverage information from them to inform the control parameter for the current study in the PPoS estimation. Adopting the notations presented in Section 2.1 Equation (2), and using the left superscript referring to the *h*‐th historical trial, we have 

(13)

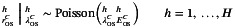


where in this context 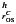 and 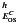 represent respectively the number of events and the total exposure time at the *final analysis* of the h‐th historical trial, non‐depending on the number of interim looks.

Following the meta‐analytic predictive approach (MAP) detailed by Roychoudhury and Neuenschwander in [23], we assume that the historical control hazard and the current control hazard are drawn from the same lognormal distribution: 

(14)

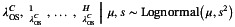


where μ represents the across trial mean parameter and s represents the between trial variability. Assuming no prior information is available for the latter parameters, the following weakly informative priors are used:

(15) μ∼Normal(0,1)s∼Half‐normal(0,0.5)

where the prior distribution for s is chosen accordingly to [23] in order to allow a wide range of heterogeneity scenarios a priori. The choice of the normal prior distribution for μ is again based on [23], and the variance parameter is arbitrarily set to reflect lack of prior information regarding the model parameter.

From the above hierarchical model, a distribution $\pi_{\lambda_{\text{OS}}^{C}}^{\text{MAP}}$ for the current control hazard can be obtained and used to inform PPoS in Equation (5). Note that only historical data are used for the construction of the MAP distribution.

Although borrowing historical information may be useful for improving the posterior estimation of the model parameters, it may still happen that concurrent data are inconsistent with historical ones. In this situation—known either as *prior‐data conflict* or *drift*—historical information should ideally be discounted, and estimation should be only driven by concurrent data. To this purpose, we use a mixture prior approach [27] which consists in combining the informative MAP prior with a vague prior in a mixture distribution. The resulting robust meta analytic prior (rMAP) takes the following form:

(16) $$\pi_{\lambda_{\text{OS}}^{C}}^{\text{rMAP}}=w_{h}\pi_{\lambda_{\text{OS}}^{C}}^{\text{MAP}}+(1-w_{h})\pi_{\lambda_{\text{OS}}^{C}}^{v}$$

where $\pi_{\lambda_{\text{OS}}^{C}}^{v}$ is the vague component of the mixture prior and $w_{h}$ is the prior weight on the informative component, reflecting the prior belief about the exchangeability between the control effect estimated from historical data and the control effect estimated from current data. Note that the term *robust* in the context of Bayesian dynamic borrowing context refers to reduced sensitivity to potential inconsistencies between the prior and the observed data, thereby yielding more reliable posterior inference.

For this approach, the weak priors from the SCA approach in Equation (12) are used for θ, γ, and $\lambda_{\text{PFS}}^{C}$.

For data borrowing on the control arm, OS data from 3 historical trials are supposed to be available with a sample size of 270 patients each, in particular the observed number of OS events 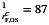, 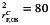, 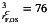, and the related total exposure times 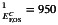, 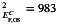, 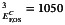 (defined as the sum of all patients' exposure times), expressed in days. Note that the data associated with the three historical trials used for control borrowing are fictitious and were generated so that the prior distribution for the control parameter $\lambda_{\text{OS}}^{C}$ match with a median OS of 8.5 months. A prior weight $w_{h}=0.9$ is used in this analysis, reflecting high confidence in the relevance of historical control data for our trial and a UIP is used as a robustification component.

### 5.2 Borrowing Historical Information From HR(PFS)‐HR(OS) Relationship

In this section, we adopt the methodology proposed by Saint‐Hilary et al. [19] and further developed by Fougeray et al. [18] in the context of GSD. This approach provides a structured Bayesian meta‐analytic framework to model the relationship between treatment effects on surrogate and primary endpoints, allowing information from historical trials to inform the estimation of the primary endpoint. The method is particularly relevant in our setting, as it enables construction of a robust surrogate prior while accounting for between‐trial variability and potential prior‐data conflicts, ensuring that the predictive inference for the primary endpoint is both informed and reliable.

Consider $H^{\prime }$ historical randomized clinical trials, where ${\hat{\gamma }}_{h^{\prime }}$ and ${\hat{\theta }}_{h^{\prime }}$ are the estimates of the true parameters $\gamma_{h^{\prime }}$ and $\theta_{h^{\prime }}$ for the treatment effects on PFS and OS respectively in each historical trial $h^{\prime }$ and are available together with their sampling variances $\delta_{h^{\prime }}$ and correlations $\rho_{h^{\prime }}$.

Referring to the methodology detailed in [19]—relying on the meta analytic approach proposed in [28]—we assume that a linear relationship holds between log(γ) and log(θ) (although the methodology may be adapted for other transformations if needed). We consider the following bi‐variate normal model:

(17) $$\left(\begin{array}{c} \log({\hat{\theta }}_{h^{\prime }})\\ \log({\hat{\gamma }}_{h^{\prime }}) \end{array}\right)∼\text{Normal}\left[\begin{array}{cc} \left(\begin{array}{c} a+b\cdot \log(\gamma_{h^{\prime }})\\ \log(\gamma_{h^{\prime }}) \end{array}\right), & \left(\begin{array}{cc} \sigma_{h^{\prime }}^{2}+\tau^{2} & \rho_{h^{\prime }}\sigma_{h^{\prime }}\delta_{h^{\prime }}\\ \rho_{h^{\prime }}\sigma_{h^{\prime }}\delta_{h^{\prime }} & \delta_{h^{\prime }}^{2} \end{array}\right) \end{array}\right]$$

The joint posterior distribution $f_{a,b,\tau }(\cdot )$ of the parameters a, b, and τ—representing respectively the intercept, the slope and the between trial variability—can be estimated via meta‐analytic regression (see [19] for details). Conditional on the regression parameters, the distribution of the treatment effect on the primary endpoint can then be obtained from the treatment effect on the surrogate endpoint as

(18) $$\log(\theta )\;|\;a,b,\tau ∼\text{Normal}\left(a+b\cdot \log(\gamma ),\tau^{2}\right)$$

We note $f_{\log(\theta )\;|\;a,b,\tau }\left(\cdot \right)$ its density. At the interim analysis, the posterior distribution of γ is estimated from the data available on the surrogate endpoint at this stage.

An informative prior distribution for the primary endpoint θ, called *surrogate prior*, is obtained by integrating Equation (18) over the joint distribution of the regression parameters as follows:

(19) $$\pi_{\log(\theta )}^{S}\left(\cdot \right)=\int f_{\log(\theta )\;|\;a,b,\tau }\left(\cdot \right)f_{a,b,\tau }\left(x,y,z\right)dxdydz$$

A *robustification* of this surrogate prior is used to handle prior‐data conflicts by combining the distribution in (19) with a vague component in a mixture distribution, and the resulting robust surrogate prior can be written as

(20) $$\pi_{\log(\theta )}^{\text{rSURR}}=w_{s}\pi_{\log(\theta )}^{S}+(1-w_{s})\pi_{\log(\theta )}^{v}$$

where $\pi_{\log(\theta )}^{v}$ is the vague component of the mixture prior and $w_{s}$ is the informative prior weight, for example, a probability measure of the prior confidence in the estimated relationship between the surrogate and the primary endpoints.

For this approach, the weak priors from the SCA approach in Equation (12) are used for γ, $\lambda_{\text{OS}}^{C}$ and $\lambda_{\text{PFS}}^{C}$.

Note that although a linear relationship between a transformation of the treatment effects on the surrogate and primary endpoints is assumed in the current formulation, the methodology is flexible and can accommodate deviations from this assumption. Specifically, the approach can be extended to incorporate any functional relationship between the parameters by modifying the mean of the marginal distribution for the treatment effect on the primary endpoint accordingly.

### 5.3 Historical Data

For data borrowing on the control arm, OS data from 3 historical trials are supposed to be available with a sample size of 270 patients each, in particular the observed number of OS events 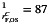, 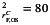, 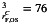, and the related total exposure times 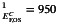, 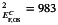, 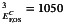 (defined as the sum of all patients' exposure times), expressed in days. Note that the data associated with the three historical trials used for control borrowing are fictitious and were generated so that the prior distribution for the control parameter $\lambda_{\text{OS}}^{C}$ matches a median OS of 8.5 months. A prior weight of $w_{h}=0.9$ is used in this analysis, reflecting strong confidence in the relevance of historical control data for our trial. A UIP is employed as the robustification component, representing a distribution whose effective sample size [29, 30] is equivalent to one, thus contributing minimal prior information.

In order to borrow information on the relationship between PFS and OS, the same Bayesian MAP used in [18] is used, fitting the model in Equation (17) with historical data on the log‐transformation of the treatment effect parameters on the two endpoints. To this end, a total of 15 randomized trials beyond the second line in mCRC were employed (12 of which taken from a systematic literature review of 2018 by Arnold [31] and 3 additional relevant trials [32, 33, 34]), evaluating both PFS and OS in similar populations (even though not testing the same drug).

**FIGURE 2:**
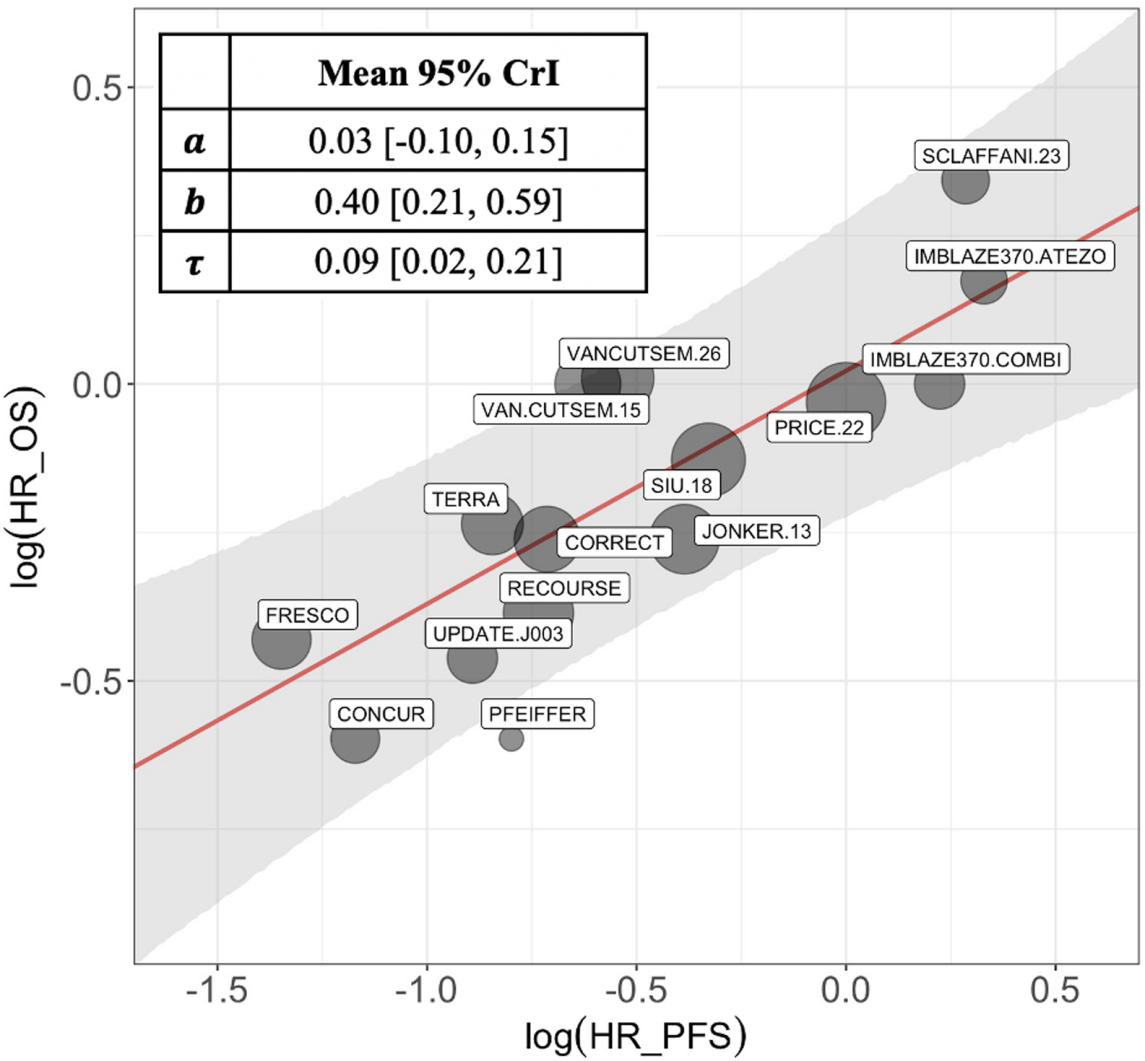
Meta‐regression to establish a log‐linear relationship between γ and θ in mCRC. In red: the regression line (with its credibility bounds in grey). The sizes of the bubbles are proportional to the inverse of the standard errors of the estimated log hazard ratio on OS.

The estimates of the hazard ratios and their variability on both PFS and OS were used to build the model in Equation (17) (Details are provided in Supporting Information). A prior weight $w_{s}=0.9$ is used in this analysis, reflecting a high confidence in the relevance of the estimated relationship estimated from historical information for our trial and a UIP is used as robustification component. The Bayesian model was fitted using 5 chains of 100 000 MCMC sampling iterations (preceded by 50.000 warm up iterations) with the R [24] package RStan [35]. We assumed a correlation coefficient $\rho_{h^{\prime }}=0.05$ between the treatment effects on the two endpoints for all the studies (see discussion in [19, 36] for more details); and improper vague prior distribution are used for the regression coefficients. The posterior medians of the regression coefficients a,b, and τ with their credibility intervals are provided in Figure 2, together with a representation of the fitted regression line.

### 5.4 Revised Simulation Results

In this simulation study, we investigate the effect of incorporating historical information in the computation of the PPoS criterion within the DCA. Two variants of the DCA are considered: the first employs a non‐informative prior distribution for the PPoS calculation (hereafter referred to as *DCA (no borrowing)*), while the second utilizes an informative prior distribution (hereafter referred to as *DCA (borrowing)*). In the latter, historical borrowing is applied both to the control parameters, as described in Section 5.1, and to the treatment effect parameters, as described in Section.

The evaluation is conducted under the same scenarios and operating characteristics (OCs) considered in the simulation study presented in Section 4. It is important to note that, in the present context, the selected scenarios acquire an interpretation in terms of alignment with *historical information*. Specifically, scenarios labeled with the identifier “0” represent alignment between the concurrent data and the meta‐analytical relationship between HR(PFS) and HR(OS), whereas scenarios labeled with the identifier “1” correspond to a situation of *prior‐data conflict* (or *drift*) between the concurrent data and the meta‐analytical relationship. Similarly, scenarios denoted by the labels “LOW” and “HIGH” indicate a *drift* between concurrent and historical control data, while a basic agreement between concurrent and historical controls is considered in scenarios A0, A1, N0, and N1. A graphical representation of the scenarios considered, with respect to their alignment with historical information, is provided in the Supporting Information.

Table 4 shows the results corresponding to the comparison between the DCA without using historical information (*DCA (no borrowing)*) and using historical information (*DCA (borrowing)*).

**TABLE 4:** Comparison between the Dual‐Criterion Approach without historical borrowing (*no borrowing*) and with historical borrowing (*borrowing*).

Scenario	Accelerated approval rate		Confirmation rate		Full approval rate		Global type I error rate	
	DCA (no borrowing)	DCA (borrowing)	DCA (no borrowing)	DCA (borrowing)	DCA (no borrowing)	DCA (borrowing)	DCA (no borrowing)	DCA (borrowing)
A0 LOW	40.9	64.1	98.8	97.7	91.3	91.3	—	—
A1 LOW	15.8	13.2	98.1	97.7	91.3	91.3	—	—
N0 LOW	0.0	0.0	—	—	1.2	1.2	1.2	1.2
N1 LOW	1.6	1.2	—	—	1.2	1.2	2.6	2.2
A0	44.1	73.0	98.2	97.5	91.1	91.1	—	—
A1	19.4	18.8	97.9	98.9	91.1	91.1	—	—
N0	0.0	0.0	—	—	1.2	1.2	1.2	1.2
N1	1.5	1.5	—	—	1.1	1.1	2.3	2.2
A0 HIGH	45.6	79.2	98.2	96.5	90.8	90.8	—	—
A1 HIGH	21.8	22.1	97.7	98.6	90.8	90.8	—	—
N0 HIGH	0.0	0.0	—	—	1.2	1.2	1.2	1.2
N1 HIGH	1.8	2.0	—	—	1.2	1.2	2.5	2.8

In the alternative scenarios, where no drift exists between the historical relationship linking HR(PFS) and HR(OS) and the concurrent data (scenarios A0‐LOW, A0, A0‐HIGH), the inclusion of historical information in the *borrowing* approach proves beneficial. In these situations, historical borrowing leads to a noticeable increase in the AA rate compared with the *no borrowing* approach (e.g., 64.1% vs. 40.9% in scenario A0‐LOW, 73.0% vs. 44.1% in scenario A0‐HIGH and 79.2% vs. 45.6% in scenario A0‐LOW).

This improvement arises because the prior informed by the historical relationship reduces uncertainty in the PPoS. Consequently, the predictive distribution of the treatment effect on the primary endpoint becomes narrower and more precise, thereby increasing the probability of meeting the PPoS criterion required for AA. However, the magnitude of the increase in AA depends on the degree of prior‐data conflict between concurrent and historical control data. For instance, an approximately 30% increase in AA is observed when no drift is present, a 35% increase when the current control outperforms the historical control, and a 25% increase when the current control underperforms relative to the historical control. The reason of this is that when the concurrent control data are superior to the historical data (scenario A0‐HIGH), the borrowing process tends to *underestimate* the control parameter, leading to an *overestimation* of the treatment effect. Conversely, when the concurrent control data are inferior to the historical data, the prior distribution on the control parameter tends to *overestimate* the control parameter, resulting in an *underestimation* of the treatment effect.

Conversely, in scenarios A1‐LOW, A1, and A1‐HIGH, where a *negative* drift is observed between the concurrent and historical data (indicating that the current treatment effect on the primary endpoint is *smaller* than that predicted by the meta‐analytic relationship for the corresponding surrogate treatment effect) the prior derived from the meta‐analytic association between HR(PFS) and HR(OS) becomes biased toward *lower* treatment effects. Consequently, under these scenarios, the advantages of incorporating the *surrogate prior* are lost, and accordingly the AA obtained with the *borrowing* approach is closely aligned with that of the *no‐borrow* approach, with only minor differences attributable to the bias introduced through historical control borrowing.

Under the partial null scenarios, and in the absence of drift between the concurrent and historical control data (scenario N1), the *borrowing* approach yields the same AA as the *no‐borrow* approach. In this case, although the surrogate prior is biased toward higher treatment effects due to discrepancies between the meta‐analytic relationship and the current data, the reduction in predictive variance achieved through borrowing is offset by the influence of the prior bias, resulting in an equivalent AA. Conversely, when the concurrent controls are inferior to the historical controls (scenario N1‐LOW), the bias introduced by the prior on the control parameter leads to a decrease in AA (1.2% vs. 1.6%). In contrast, when the concurrent controls are superior to the historical controls (scenario N1‐HIGH), the same mechanism results in an inflation of AA (2.0% vs. 1.8%). Under the double null scenarios (N0‐LOW, N0, N0‐HIGH), both approaches produce identical results, with an AA equal to zero.

As expected, both approaches yield identical results in terms of FA, since this metric depends solely on the analysis of the primary endpoint within the concurrent data, which is identical across methods. Consequently, any differences in G‐t1E between the *borrowing* and *no‐borrow* approaches arise exclusively from differences in their respective AA rates. Accordingly, in scenarios where a decrease in AA is observed, a corresponding reduction in G‐t1E is also noted (e.g., from 2.6% to 2.2% in scenario N1‐LOW). Conversely, in scenarios where an increase in AA occurs, a corresponding inflation in G‐t1E is observed (e.g., from 2.5% to 2.8% in scenario N1‐HIGH).

In terms of CR, *borrowing* and *no borrowing* display comparable performance, with only minor variations observed across scenarios. Both approaches demonstrate a high likelihood that treatments granted AA would ultimately confirm efficacy at the FA stage, indicating overall robustness of the dual‐criterion design.

Overall, the comparison between *borrowing* and *no borrowing* highlights the trade‐off between stability and efficiency. The *no borrowing* approach, relying exclusively on concurrent data, provides consistent but conservative estimates, while the *borrowing* approach, through the use of historical and surrogate information, enhances decision‐making power and improves AA rates under concordant conditions. However, the latter's performance depends critically on the compatibility between historical and current evidence, as greater inconsistency can attenuate its benefits or even reduce accuracy in decision‐making.

## 6 Sensitivity Analysis

### 6.1 Motivation

When evaluating a given trial design, health authorities typically request an assessment of the *frequentist* OCs under pre‐specified scenarios, such as the null scenario for type I error control and the alternative scenario for power evaluation. However, particularly in settings where historical borrowing is incorporated into the design, it is a standard regulatory requirement [37, 38] to report frequentist OCs under additional scenarios that may deviate from both the design assumptions and the historical data sources. These evaluations are used by regulators to assess the robustness of the design with respect to potential violations of the assumptions underlying the use of external information.

It is acknowledged, however, that not all scenarios are equally plausible [39]. For instance, a scenario assuming no treatment effect on the primary endpoint but a very large effect on the surrogate endpoint (e.g., γ=0.2, θ=1) is less plausible than the double null scenario (γ=1, θ=1), particularly if the surrogacy assumption between PFS and OS holds. Similarly, observing a median OS of 2 months for the current control arm is less credible than observing a median OS of 8 months, given that a median OS of 8.5 months is assumed by design.

For this reason, Best et al. [39] advocate for the use of appropriate Bayesian metrics when evaluating Bayesian designs. These metrics involve averaging the frequentist OCs, each computed under a specific configuration of true parameters, over a so‐called *design prior*, which represents a prior distribution reflecting the relative plausibility of different parameter values. In this framework, each scenario contributes to the overall metric proportionally to its plausibility, as defined by the design prior. As a result, a high type I error rate under an implausible scenario has a limited impact on the overall evaluation metric, whereas the same error rate under the design assumption would contribute more significantly.

Note that, differently from the *analysis prior*—which synthesizes all available information regarding the model parameter and is employed in the actual analysis of the trial—the *design prior* represents an assumption regarding the distribution of the true parameters and is uniquely used for design evaluation (hence may or may not be consistent with the prior knowledge regarding the true parameter).

In this section, an extensive simulation study is conducted to (i) assess the frequentist OCs of the proposed approaches across a *continuous* range of possible scenarios, thereby enabling a *quantitative* evaluation of the impact of prior‐data conflict on these OCs, and (ii) examine several *global* properties of the Bayesian methods, including the Bayesian OCs introduced by Best et al. [39], to gain a deeper understanding of the trade‐off between the overall benefits and risks associated with the Bayesian designs under consideration.

We acknowledge that, in current practice, the assessment of design performance across different levels of prior‐data conflicts is predominantly qualitative. For example, Type I error control is commonly evaluated by inspecting a grid of simulated scenarios and visually identifying regions in which inflation may occur. To this matter, it is important to specify that these global metrics should be viewed as means of *summarizing* the OCs exhibited across this simulation grid. These quantities are not intended to replace qualitative evaluation; rather, they serve as complementary measures that *enhance* and inform the decision‐making process.

### 6.2 Setting

Two analyses are performed: in the first, effective treatments are tested under the alternative hypothesis θ = 0.71; in the second, noneffective treatments are tested under the null hypothesis θ = 1. For both analyses, the OCs are simulated in a 20 by 20 grid of scenarios, which are set by varying log(γ) and median OS for concurrent controls in the set of equispaced values in Γ×Λ, where Γ=[−2,0.5] and Λ=[3,16] (months). The transformation of Λ on the hazard scale $\Lambda^{*}=\frac{\log\left(2\right)}{\Lambda }$ (following from the assumption of exponentially distributed $\lambda_{\text{OS}}^{C}$) will be used to derive the prior distribution for the control parameter. The extreme values of the simulation grid are chosen considering that

- All scenarios where the median OS for concurrent controls is lower than 2.1 months correspond to situations in which average PFS is longer than OS (which is impossible since all OS events (deaths) are also PFS events);
- Values greater than 16 months represent very unlikely scenarios in that disease (we remind that a median OS for concurrent control of 8.5 months was assumed by design);
- For γ, the extreme values correspond to hazard ratios of 0.135 and 1.65, which are considered the thresholds below (respectively, above) which it is implausible to observe values (we remind that a treatment effect for the surrogate endpoint γ=0.525 was assumed by design).

In order to understand the impact of the prior weights in the proposed approaches, the above analyses are performed under different choices of $w_{h}$ and $w_{s}$ in the set 𝒲=(0.1,0.3,0.5,0.7,0.9).

### 6.3 Choice of Design Priors

In the context of this work, two distinct types of historical borrowing are considered: one on the hazard parameter for the control arm, $\lambda_{\text{OS}}^{C}$, and the other on the treatment effect on the primary endpoint, θ. Consequently, *design priors* must be specified for both parameters.

For the historical control parameter $\lambda_{\text{OS}}^{C}$, we use the MAP prior $\pi_{\lambda_{\text{OS}}^{C}}^{\text{MAP}}$ described in Section 5.1 as design prior, meaning that the distribution of assumed values for the control hazard employed for design evaluation is consistent to the prior assumption about the control parameter used at the analysis stage. This choice is motivated by the fact that $\pi_{\lambda_{\text{OS}}^{C}}^{\text{MAP}}$ represents the most plausible assumption on the control parameter so far.

On the other hand, historical data introduced in Section 5.4—even though representing treatment effects—only inform the *relationship* between γ and θ, but they provide no direct information on the treatment effect of the concurrent treatment. A modification of the procedure described in Section 5.2 can be used in order to determine a *design prior* for the parameter γ.

Let suppose that a dual representation of the bi‐variate model in Equation (17) holds:

(21) $$\left(\begin{array}{c} \log({\hat{\gamma }}_{h^{\prime }})\\ \log({\hat{\theta }}_{h^{\prime }}) \end{array}\right)∼\text{Normal}\left[\begin{array}{cc} \left(\begin{array}{c} ã+\tilde{b}\cdot \log(\theta_{h^{\prime }})\\ \log(\theta_{h^{\prime }}) \end{array}\right), & \left(\begin{array}{cc} \sigma_{h^{\prime }}^{2}+{\tilde{\tau }}^{2} & \rho_{h^{\prime }}\sigma_{h^{\prime }}\delta_{h^{\prime }}\\ \rho_{h^{\prime }}\sigma_{h^{\prime }}\delta_{h^{\prime }} & \delta_{h^{\prime }}^{2} \end{array}\right) \end{array}\right]$$

where in this case the marginal relative to the treatment effect parameter on the surrogate endpoint is expressed in terms of the treatment effect on the primary endpoint. Once posterior distributions for the regression parameters ã, $\tilde{b}$ and $\tilde{\tau }$ are obtained, a distribution for log(γ) conditional on the regression coefficients takes the following form

(22) $$\log(\gamma )\;|\;ã,\tilde{b},\tilde{\tau }∼\text{Normal}\left(ã+\tilde{b}\cdot \log\left(\theta^{\#}\right),{\tilde{\tau }}^{2}\right)$$

where $\theta^{\#}$ may represent the most likely treatment effect on the primary endpoint (e.g., the alternative hypothesis). The *design prior* for log(γ) is finally obtained by marginalising the conditional distribution in Equation (22) over the joint distribution of the regression parameters:

(23) $$p_{\log(\gamma )}\left(\cdot \right)=\int f_{\log(\gamma )\;|\;ã,\tilde{b},\tilde{\tau }}\left(\cdot \right)f_{ã,\tilde{b},\tilde{\tau }}\left(x,y,z\right)dxdydz.$$

Note that $\theta^{\#}$ is a fixed value in this context; however a *design prior* distribution for $\theta^{\#}$ can be used in principle, even though it would make the derivation of the design prior for log(γ) more complex.

### 6.4 Evaluation Metrics

For the current analysis first the G‐t1E will be evaluated for each point of the grid in order to analyze the impact of median(OS) for concurrent controls and the treatment effect on the surrogate endpoint log(γ) on the false positive rate. Moreover, three different global metrics are proposed:

- Maximum Global type I Error Rate, defined as the maximum probability to pass either AA Analysis or FA analysis under the null hypothesis of no treatment effect on the primary endpoint, among the scenarios simulated in the grid.
(24) max(G‐t1E)=maxx∈Γy∈Λ∗[G‐t1E(x,y)]
- Average Global type I error rate, defined as the probability to pass either AA Analysis or FA analysis under the null hypothesis of no treatment effect on the primary endpoint, averaged over the *design priors* defined in Section 6.3:
(25) $$\text{avg(G‐t1E)}=\int_{\Lambda^{*}}\int_{\Gamma }\text{G‐t1E}\left(x,y\right)\cdot \pi_{\lambda_{\text{OS}}^{C}}^{\text{MAP}}(y)p_{\log(\gamma )}(x)dxdy$$
- Average Accelerated Approval Power, defined as the probability to pass AA Analysis under the alternative hypothesis, averaged over the *design priors* defined in Section 6.3:
(26) $$\text{avg(AA‐Pow)}=\int_{\Lambda^{*}}\int_{\Gamma }\text{AA‐Pow}(x,y)\cdot \pi_{\lambda_{\text{OS}}^{C}}^{\text{MAP}}(y)p_{\log(\gamma )}(x)dxdy$$

where $\text{G‐t1E}\left(x,y\right)=\mathbb{P}\left[\text{AA}\cup \text{FA}\;|\;\gamma =x,\;\lambda_{\text{OS}}^{C}=y,\;\theta =1\right]$ and $\text{AA‐Pow}\left(x,y\right)=\mathbb{P}\left[\text{AA}\;|\;\gamma =x,\;\lambda_{\text{OS}}^{C}=y,\;\theta =0.71\right]$.

For the computation of the bi‐variate integrals in Equations (25) and (26): a kernel estimation of the density functions corresponding to the design priors is obtained (using the function *density* of R [24]); G‐t1E(x,y) and AA‐Pow(x,y) are estimated by the proportion of trials meeting the criteria out of 3000 simulated trials in the grid of scenarios; and the trapezoid rule is employed in order approximate the integrals over the grid.

### 6.5 Results

**FIGURE 3:**
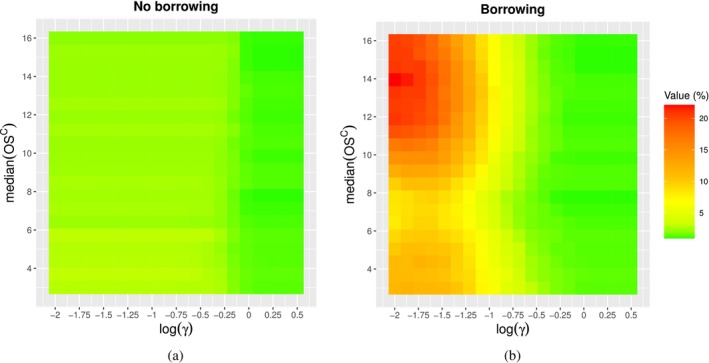
Global type I error rate under different pairs [log(γ), median(${\text{OS}}^{C}$)] in the simulation grid. Prior weights for historical borrowing on the concurrent control parameter $\lambda_{\text{OS}}^{C}$ and the surrogate treatment effect γ are set to $w_{h}=0.9$ and $w_{s}=0.9$.

Figure 3 illustrates how the global type I error rate varies as a bivariate function of the logarithm of the surrogate treatment effect (x‐axis) and the median OS in the control arm (y‐axis). Under the assumption of an exponential distribution for OS, the median OS is directly related to the control hazard rate $\lambda_{\text{OS}}^{C}$ through the relationship $\text{median(OS)}=\log(2)/\lambda_{\text{OS}}^{C}$. The choice of representing the y‐axis in terms of the median OS, rather than the hazard rate, is intended to enhance interpretability for applied readers. The heatmap uses color shading to represent the same quantity, that is, the simulated global type I error rate at each combination of surrogate effect and median OS. Namely, green regions of the space represent values of G‐t1E equal or below the nominal level, while from yellow to red regions represent increasing levels of the latter metric. An equivalent figure displaying the Accelerated Approval power (AA‐Pow) is presented in the Supporting Information.

Concerning the *no borrowing* approach, variations in the median OS of the current control arm exert only a minor influence on the G‐t1E, which remains approximately 2.5% across all scenarios considered. A slight decrease in G‐t1E is observed when γ≈0 as the median control OS increases. In this setting, higher control‐arm survival results in a greater number of PFS events being available at the interim analysis, thereby enhancing the estimation of the surrogate treatment effect, γ, and consequently reducing the risk of an incorrect A. It should be noted that this pattern manifests only when the surrogate treatment effect is small, as a larger number of observed PFS events can meaningfully improve the estimation. For higher surrogate treatment effects, the PFS criterion is consistently satisfied, rendering this effect negligible. Since no historical data are utilized to estimate the relationship between surrogate and primary endpoints, the PPoS criterion remains unaffected by log(γ). Consequently, variations of G‐t1E along the x‐axis are confined to values near log(γ)≈0, with G‐t1E approaching zero for γ>0.

Regarding the *borrowing* approach, distinct patterns emerge in G‐t1E, as summarized below:

– Similar to the *no borrowing* approach, G‐t1E approaches zero when γ>0. This behavior arises from the PFS criterion, which is consistently unmet in the absence of a treatment effect on the surrogate endpoint.
– The influence of borrowing information on the control parameter is observed along the Y‐axis: for a given value of γ, when the current control is superior (median OS >8.5 months) relative to historical controls, G‐t1E increases, reaching a maximum when the median OS of the concurrent control is approximately 13 months. Conversely, for inferior current controls, G‐t1E decreases, attaining a minimum around a median OS of 7 months. Beyond these thresholds, the impact of prior‐data conflict is mitigated by the robust component of the mixture prior, resulting in a decrease in G‐t1E for median OS exceeding 13 months and an increase in G‐t1E for median OS below 8 months.
– The influence of borrowing information on the control parameter is also observed along the X‐axis: for a fixed value of ${\text{median(OS}}^{C})$, when the drift between concurrent data and the meta‐analytic relationship between HR(PFS) and HR(OS) is small (−0.5<log(γ)<0), G‐t1E is reduced due to the increased precision afforded by the surrogate prior. In contrast, as prior‐data conflict increases (γ<−0.75), G‐t1E inflates, reaching a maximum around log(γ)=−1.75. A subsequent decrease in G‐t1E for more extreme values of γ is attributable to the robust component of the mixture prior, which downweights prior information in the presence of substantial drift.

Since the OCs within the *borrowing* approach exhibit substantial variations across the parameter space, the assessment of *global* metrics, as proposed in Section 6.4, is convenient in the assessment of benefits and risks of Bayesian designs, particularly with respect to the choice of the borrowing parameters.

In the Supporting Information, the maximum G‐t1E obtained under different choices of the mixture weights $w_{h}$ and $w_{s}$ is presented. The results indicate that higher mixture weights are associated with an increase in the maximum G‐t1E. Specifically, when both weights are small (e.g., $w_{h}=1$, $w_{s}=1$), the maximum G‐t1E is approximately 4%, whereas for larger weights (e.g., $w_{h}=0.9$, $w_{s}=0.9$), the maximum G‐t1E exceeds 20%. Notably, the mixture weight corresponding to the surrogate prior ($w_{s}$) exerts a stronger influence on the maximum G‐t1E.

**FIGURE 4:**
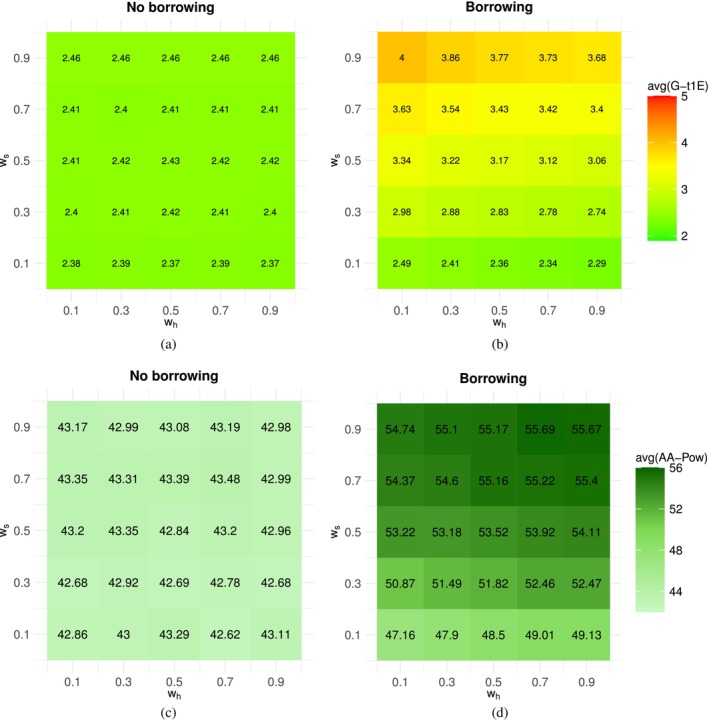
(Top row) Average Global type I error avg(G‐t1E) computed for different pairs of the prior mixture weights $\left(w_{h},w_{s}\right)$. (Bottom row) Average Accelerated Approval Power avg(AA‐Pow) computed for different pairs of the prior mixture weights $\left(w_{h},w_{s}\right)$. In both cases, the set of weights is 𝒲=(0.1,0.3,0.5,0.7,0.9).

Figure 4 (panels (a) and (b)) displays the average G‐t1E, denoted as avg(G‐t1E), for both the *no borrowing* and *borrowing* approaches. For the former, this metric remains at the nominal level of 2.5%, with no variation across different pairs of $\left(w_{h},w_{s}\right)$, as no information borrowing is implemented. For the latter, an increase in avg(G‐t1E) is observed with increasing values of $w_{s}$ (ranging from 2.5% to approximately 4%), while a slight decrease is noted as $w_{h}$ increases. Notably, although a mild inflation relative to the nominal level is detected, the avg(G‐t1E) remains relatively low. This outcome reflects the fact that although large values of G‐t1E are possible, the highest increases in G‐t1E occur in regions of the parameter space that have low probability under the *design prior* employed, thus only slightly impact the averaging process.

Figure 4 (panels (c) and (d)) presents the average Accelerated Approval power, denoted as avg(AA‐pow), for both the *no borrowing* and *borrowing* approaches. In the *no borrowing* case, avg(AA‐pow) is approximately 43%, with no variation across different combinations of $\left(w_{h},w_{s}\right)$, consistent with the absence of information borrowing. Conversely, under the *borrowing* approach, avg(AA‐pow) increases with larger values of $w_{h}$ and $w_{s}$, reaching up to 65% when strong borrowing is applied ($w_{h}=w_{s}=0.9$), while lower values (around 47%) are observed under minimal borrowing ($w_{h}=w_{s}=0.1$). These results emphasize the added value of incorporating historical borrowing in enhancing the evidence supporting an AA request.

In summary, borrowing information within the dual‐criterion framework entails both benefits and risks. Strong borrowing is associated with higher average AA rates under the alternative scenario compared with the *no borrowing* approach; however, it also carries certain risks, particularly in terms of increases in both the maximum global type I error and the inflation of the average global type I error rate. While we acknowledge the importance of evaluating standard OCs under fixed scenarios, we advocate that the assessment of Bayesian metrics represents a valuable tool for quantifying the benefits and risks of Bayesian designs, thus ultimately facilitating informed discussions between sponsors and regulators.

## 7 Discussion

In recent decades, the increasing need to deliver effective treatments for life‐threatening diseases has led various health authorities to implement AA pathways, allowing promising treatments to enter the market earlier when sufficient evidence supports their efficacy.

In this context, we proposed a novel approach for AA interim analyses within phase III GSD. This approach tests treatment efficacy on a short‐term surrogate endpoint alongside the predictive probability of study success (PPoS) on the long‐term primary endpoint. Different strategies are proposed to inform PPoS by (i) leveraging historical data on the control arm and (ii) borrowing information from a documented relationship between surrogate and primary endpoints, derived from meta‐regression on historical trials. For historical control borrowing, we employed the methodology described in [23]. For incorporating historical information on the endpoints' relationship, we applied the methods in [18, 19], where an informative prior (surrogate prior) for the primary endpoint, derived by combining partial surrogate endpoint data and regression parameters, is updated with primary endpoint data available at the interim analysis.

Numerical results indicate that reinforcing efficacy testing on the surrogate endpoint through a predictive criterion based on the PPoS within the DCA reduces the probability of requesting AA for treatments with no true efficacy. However, this improvement is associated with a reduction in the probability of meeting the criteria for an AA request, which may be less favorable from a sponsor's perspective.

Within the dual‐criterion framework, incorporating historical information improves performance by maintaining a high probability of satisfying AA criteria when concurrent and historical data are consistent, while also keeping a low probability of requesting AA for ineffective treatments. These findings are supported by the assessment of Bayesian operating characteristics proposed by Best et al. [39].

To address potential prior‐data conflict in both historical sources, we applied a robust mixture approach as presented in [27]. This approach requires specification of a prior weight reflecting the relevance of historical data for the current trial. We conducted a comparative analysis of OCs under different prior weight choices, which may guide decision‐making. Alternative methods for determining prior weights include empirical Bayes approaches [40, 41]. Other methodologies for incorporating historical data, such as power priors [42], commensurate priors [20], or elastic priors [43], could also be considered within this framework.

A conservative choice for the decision thresholds was applied to ensure that the global type I error under the double null scenario is controlled independently of the threshold for the predictive criterion, providing additional protection under a partial null scenario. Consequently, the global type I error remains below the nominal level. Less conservative options may be explored, for example, by selecting the PPoS threshold based on maintaining the global type I error under the double null scenario. In our proposal, an equal allocation of the nominal type I error rate was used for the FA analyses and the PFS criterion in AA analyses. Alternative allocations may be appropriate, such as emphasizing the early AA analysis when the surrogate endpoint is considered highly predictive, or prioritizing the FA analysis to reduce the risk of incorrect AA decisions.

Although our methodology jointly evaluates efficacy on two endpoints, it is inherently univariate, meaning that potential patient‐level correlations between surrogate and primary endpoints are not explicitly incorporated, and the data are treated as independent. This assumption is reasonable in settings where surrogate outcomes from phase II inform future phase III trials [19]. In our context, however, if patient‐level data are available, within‐trial correlation could be estimated using concurrent data at the interim analysis. Simulations with correlated datasets indicate that when historical borrowing informs PPoS in the dual‐criterion framework, the impact of correlation is minimal. Without historical borrowing, moderate increases in the AA rate were observed under alternative scenarios, suggesting that the independence assumption is conservative.

The methodology presented is based on probabilistic assessments of treatment differences, but the clinical relevance of observed hazard ratios should also be discussed with regulatory authorities, considering the clinical context and patient population.

The proposed methodology should be interpreted primarily as a supportive framework for sponsors in guiding internal decisions regarding AA applications. Final decisions regarding AA remain with regulatory authorities and are based on multiple factors, including safety and efficacy. Strengthening evidentiary criteria for AA may help sponsors reduce the risk of subsequent withdrawal of approval, while assisting regulators in avoiding premature commercialization of treatments with uncertain efficacy. The PPoS criterion could potentially serve as an additional tool for regulatory evaluation: a high interim PPoS may inform post‐marketing commitments, while a moderate PPoS may indicate the need for stricter evidentiary requirements before FA.

In conclusion, although our work uses a Bayesian framework, the methodology can be adapted to a frequentist setting. This could involve standard hypothesis tests (e.g., log‐rank test) with predictive probability replaced by a frequentist analogue, such as predictive or conditional power. Incorporating historical information in a frequentist framework requires methods that preserve statistical validity, such as test‐then‐pool strategies or hierarchical models that account for heterogeneity while maintaining type I error control.

## Funding

This work was supported by the National Institute for Health and Care Research (Grant No. NIHR300576), the Institut de Recherches Servier, and the Department of Health Care Services (Grant No. MC UU 00040/03).

## Conflicts of Interest

The authors declare no conflicts of interest.

## Supporting information




**Data S1**: sim70361‐sup‐0001‐Supinfo.pdf.

## Data Availability

The authors have nothing to report.
