# Supplementary material for: Dual‐Criterion Approach Incorporating Historical Information to Seek Accelerated Approval With Application in Time‐to‐Event Group Sequential Trials
Source: Stat Med. 2026 Jan 23;45(1-2):e70361. doi: 10.1002/sim.70361 (PMC12828486; doi:10.1002/sim.70361)
Supplement: Supplementary file 1 — Data S1: sim70361‐sup‐0001‐Supinfo.pdf. [file SIM-45-0-s001.pdf]

Supplementary Material

for the article:

"Dual-criterion approach incorporating historical information to seek accelerated approval with application in time-to-event group sequential trials"

Marco Ratta[1], Gaëlle Saint-Hilary[2], Valentine Barboux[3], Mauro Gasparini[1], Donia Skanji[3] and Pavel Mozgunov[4,2]

[1]Department of Mathematical Sciences, Polytecnic University of Turin, Italy

[2]Servier National Research Institute (IRIS), France

[3]Department of statistical methodology, Saryga, France

[4]MRC Biostatistics Unit, University of Cambridge, United Kingdom

1 Additional Tables

Table S1: Comparison between single-criterion approach (SCA) and dual-criterion approach (DCA). Patient level correlation between PFS and OS is set to 0.45.

Scenario	Accelerated Approval Rate		Confirmation Rate		Full Approval Rate		Global type I Error Rate	
	SCA	DCA (no borrow)	SCA	DCA (no borrow)	SCA	DCA (no borrow)	SCA	DCA (no borrow)
A0 LOW	100	43.4	90.4	98.4	90.4	90.4	–	–
A1 LOW	40.1	25.0	95.0	98.8	90.4	90.4	–	–
N0 LOW	1.7	0.0	–	–	1.1	1.1	2.8	1.1
N1 LOW	96.1	1.7	–	–	1.1	1.1	96.1	2.6
A0	100	45.1	90.7	98.2	90.7	90.7	–	–
A1	44.0	27.1	96.1	99.3	90.7	90.7	–	–
N0	1.3	0.1	–	–	1.0	1.0	2.3	1.1
N1	97.0	1.4	–	–	1.0	1.0	97.0	2.2
A0 HIGH	100	47.0	90.9	98.7	90.9	90.9	–	–
A1 HIGH	46.8	28.7	94.7	99.7	90.9	90.9	–	–
N0 HIGH	1.5	0.3	–	–	1.2	1.2	2.7	1.5
N1 HIGH	98.6	1.5	–	–	1.2	1.2	98.6	2.4

Table S2: Comparison between the Dual-Criterion Approach without historical borrowing (*no borrow*) and with historical borrowing (*borrow*).Patient level correlation between PFS and OS is set to 0.45.

Scenario	Accelerated Approval Rate		Confirmation Rate		Full Approval Rate		Global type I Error Rate	
	DCA (no borrow)	DCA (borrow)	DCA (no borrow)	DCA (borrow)	DCA (no borrow)	DCA (borrow)	DCA (no borrow)	DCA (borrow)
A0 LOW	43.4	64.5	98.4	97.4	90.4	90.4	–	–
A1 LOW	25.0	20.8	98.8	98.6	90.4	90.4	–	–
N0 LOW	0.0	0.0	–	–	1.1	1.1	2.8	1.1
N1 LOW	1.7	1.3	–	–	1.1	1.1	2.6	2.2
A0	45.1	73.1	98.2	97.1	90.7	90.7	–	–
A1	27.1	25.6	99.3	99.6	90.7	90.7	–	–
N0	0.1	0.0	–	–	1.0	1.0	1.1	1.0
N1	1.4	1.6	–	–	1.0	1.0	2.2	2.4
A0 HIGH	47.0	77.9	98.7	96.8	90.9	90.9	–	–
A1 HIGH	28.7	29.2	97.7	99.7	90.9	90.9	–	–
N0 HIGH	0.3	0.0	–	–	1.2	1.2	1.5	1.2
N1 HIGH	1.5	2.1	–	–	1.2	1.2	2.4	3.0

Table S3: Comparison between the Dual-Criterion Approach without historical borrowing (*no borrow*) and with historical borrowing (*borrow*) under the double null scenario ($\gamma = 1, \theta = 1$). Different informative components have been used for $\log(\theta)$ within the Robust Mixture Prior (all with $w_S = 0.9$), namely $\mathcal{N}(-1.61, 1/100)$ (average HR=0.2), $\mathcal{N}(-0.92, 1/100)$ (average HR=0.4), $\mathcal{N}(-0.51, 1/100)$ (average HR=0.6), $\mathcal{N}(-0.22, 1/100)$ (average HR=0.8) and $\mathcal{N}(0, 1/100)$ (average HR=1).

Informative Component of the RMP used for $\log(\theta)$	PFS Criterion		PPoS Criterion		Accelerated Approval Rate		Full Approval Rate		Global type I Error Rate	
	DCA (no borrow)	DCA (borrow)	DCA (no borrow)	DCA (borrow)	DCA (no borrow)	DCA (borrow)	DCA (no borrow)	DCA (borrow)	DCA (no borrow)	DCA (borrow)
$\mathcal{N}(-1.61, 1/100)$	1.2	1.2	1.3	0.9	0.0	0.0	1.2	1.2	1.2	1.2
$\mathcal{N}(-0.92, 1/100)$	1.2	1.2	1.3	5.2	0.0	0.0	1.2	1.2	1.2	1.2
$\mathcal{N}(-0.51, 1/100)$	1.2	1.2	1.3	17.0	0.0	0.1	1.2	1.2	1.2	1.3
$\mathcal{N}(-0.22, 1/100)$	1.2	1.2	1.3	0.4	0.0	0.0	1.2	1.2	1.2	1.2
$\mathcal{N}(0, 1/100)$	1.2	1.2	1.3	0.0	0.0	0.0	1.2	1.2	1.2	1.2

Table S4: Clinical trials included in the Meta-Analytic model

**Clinical Trials Included in the Meta-Analytic Model**	**HR(OS) [95% CI]**	**HR(PFS) [95% CI]**
TERRA [12]	0.79 [0.62; 0.99]	0.43 [0.34; 0.54]
PFEIFFER [8]	0.55 [0.32; 0.94]	0.45 [0.29; 0.72]
RECOURSE [5]	0.68 [0.58; 0.81]	0.48 [0.41; 0.57]
UPDATE.J003 [13]	0.63 [0.45; 0.87]	0.41 [0.28; 0.59]
CORRECT [3]	0.77 [0.64; 0.94]	0.49 [0.42; 0.58]
CONCUR [7]	0.55 [0.40; 0.77]	0.31 [0.22; 0.44]
VAN.CUTSEM.15 [1]	1.00 [0.82; 1.22]	0.54 [0.44; 0.66]
JONKER.13 [4]	0.77 [0.64; 0.92]	0.68 [0.57; 0.80]
SIU.18 [11]	0.88 [0.74; 1.03]	0.72 [0.62; 0.84]
PRICE.22 [9]	0.97 [0.84; 1.11]	1.00 [0.88; 1.14]
SCLAFFANI [10]	1.41 [0.99; 2.00]	1.33 [0.98; 1.83]
VANCUTSEM [1]	1.01 [0.86; 1.19]	0.58 [0.49; 0.69]
FRESCO [6]	0.65 [0.51; 0.83]	0.26 [0.21; 0.34]
IMBLAZE370.COMBI [2]	1.00 [0.73; 1.38]	1.25 [0.94; 1.65]
IMBLAZE370.ATEZO [2]	1.19 [0.83; 1.71]	1.39 [1.00; 1.94]

2 Additional Figures

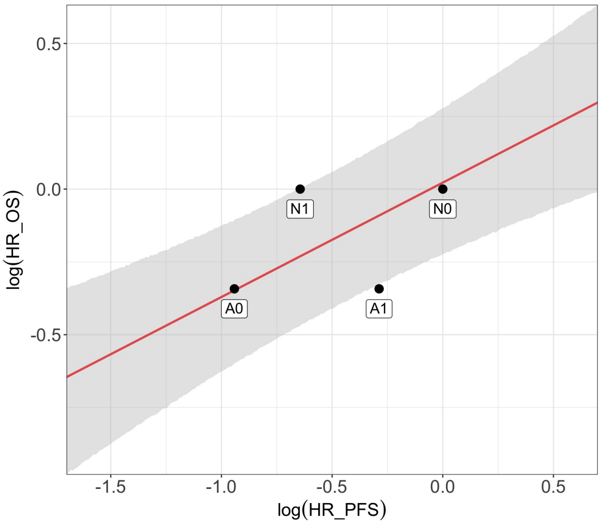

Figure S1: Graphical representation of the considered scenarios with respect to the meta-analytic regression line. Scenarios close to the red line (A0 and N0) are in accordance with the historical information, scenarios far from the red line (A1, N1) are in conflict with historical information.

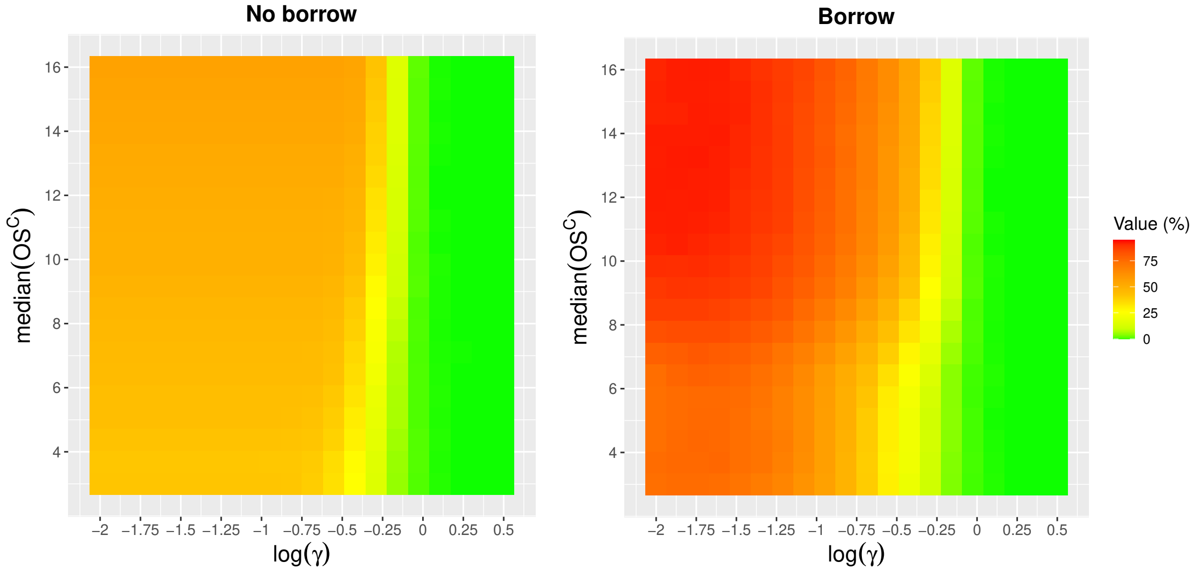

(a) (b)

Figure S2: Accelerated Approval Power (AA-Pow) under different pairs [log(γ), median(OS^{C})] in the simulation grid. Prior weights for historical borrowing on the concurrent control parameter $\lambda_{\mathrm{OS}}^{C}$ and the surrogate treatment effect γ are set to $w_h = 0.9$ and $w_s = 0.9$.

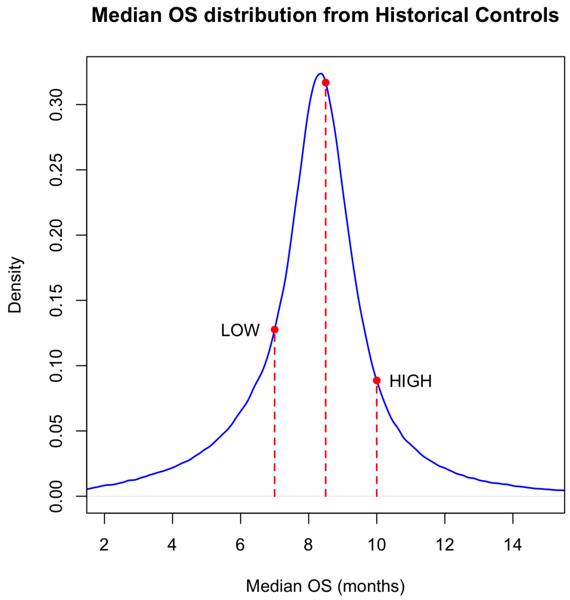

Figure S3: Graphical representation of the considered scenarios with respect to the meta-analytic distribution of the median OS from the historical control informations. Scenarios referred with "LOW" present a lower median OS with respect to the one expected from the historical MAP while scenarios referred with "HIGH" present an higher median OS with respect to the one expected from the historical MAP. Scenarios with no label do not present relevant drift in median OS with respect to the historical MAP.

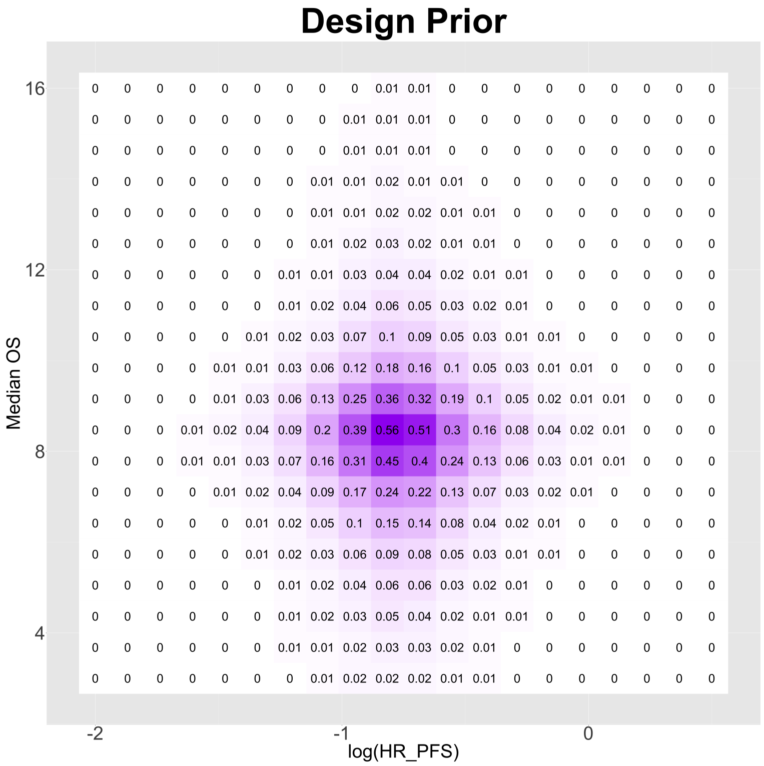

Figure S4: Graphical representation of the bi-variate Design prior density on the simulation grid.

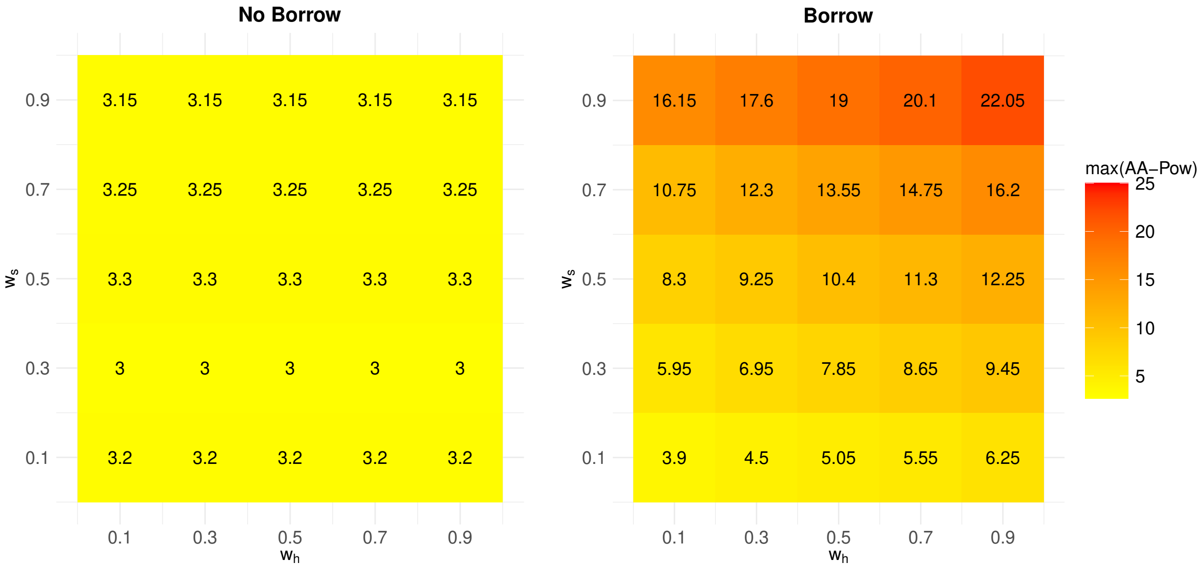

(a) (b)

Figure S5: Maximum Accelerated Approval Power max(AA-Pow) computed for different pairs of the prior mixture weights (w_h, w_s) in the set $\mathcal{W} = (0.1, 0.3, 0.5, 0.7, 0.9)$

References

[1] E. Van Cutsem, T. Yoshino, H.-J. Lenz, S. Lonardi, A. Falcone, M.L. Limon, M.P. Saunders, A. Sobrero, E. Maiello, Y.S. Park, R. Ferreiro Monteagudo, Y.S. Hong, J. Tomasek, H. Taniguchi, F. Ciardiello, J. Hocke, Z. Oum'hamed, S. Vlassak, M. Studeny, and J. Tabernero. gastrointestinal tumours, colorectal nintedanib plus best supportive care (bsc) versus placebo plus bsc for the treatment of patients (pts) with colorectal cancer (crc) refractory to standard therapies: Results of the phase iii lume-colon 1 study. *Annals of Oncology*, 27:vi559, 10 2016. DOI: 10.1093/annonc/mdw435.12.

[2] Cathy Eng, Tae Won Kim, Johanna Bendell, Guillem Argilés, Niall C Tebbutt, Maria Di Bartolomeo, Alfredo Falcone, Marwan Fakih, Mark Kozloff, Neil H Segal, Alberto Sobrero, Yibing Yan, Ilsung Chang, Anne Uyei, Louise Roberts, Fortunato Ciardiello, and IMblaze370 Investigators. Atezolizumab with or without cobimetinib versus regorafenib in previously treated metastatic colorectal cancer (imblaze370): a multicentre, open-label, phase 3, randomised, controlled trial. *The Lancet. Oncology*, 20:849–861, 6 2019. DOI: 10.1016/S1470-2045(19)30027-0.

[3] Axel Grothey, Eric Van Cutsem, Alberto Sobrero, Salvatore Siena, Alfredo Falcone, Marc Ychou, Yves Humblet, Olivier Bouché, Laurent Mineur, Carlo Barone, Antoine Adenis, Josep Tabernero, Takayuki Yoshino, Heinz-Josef Lenz, Richard M Goldberg, Daniel J Sargent, Frank Cihon, Lisa Cupit, Andrea Wagner, Dirk Laurent, and CORRECT Study Group. Regorafenib monotherapy for previously treated metastatic colorectal cancer (correct): an international, multicentre, randomised, placebo-controlled, phase 3 trial. *Lancet (London, England)*, 381:303–12, 1 2013. DOI: 10.1016/S0140-6736(12)61900-X.

[4] Derek J Jonker, Chris J O'Callaghan, Christos S Karapetis, John R Zalcberg, Dongsheng Tu, Heather-Jane Au, Scott R Berry, Marianne Krahn, Timothy Price, R John Simes, Niall C Tebbutt, Guy van Hazel, Rafal Wierzbicki, Christiane Langer, and Malcolm J Moore. Cetuximab for the treatment of colorectal cancer. *The New England journal of medicine*, 357:2040–8, 11 2007. DOI: 10.1056/NEJMoa071834.

[5] Mario M Leitao, Usha S Kreaden, Vincent Laudone, Bernard J Park, Emmanouil P Pappou, John W Davis, David C Rice, George J Chang, Emma C Rossi, April E Hebert, April Slee, and Mithat Gonen. The recourse study: Long-term oncologic outcomes associated with robotically assisted minimally invasive procedures for endometrial, cervical, colorectal, lung, or prostate cancer: A systematic review and meta-analysis. *Annals of surgery*, 277:387–396, 3 2023. DOI: 10.1097/SLA.0000000000005698.

[6] Jin Li, Shukui Qin, Rui-Hua Xu, Lin Shen, Jianming Xu, Yuxian Bai, Lei Yang, Yanhong Deng, Zhen-Dong Chen, Haijun Zhong, Hongming Pan, Weijian Guo, Yongqian Shu, Ying Yuan, Jianfeng Zhou, Nong Xu, Tianshu Liu, Dong Ma, Changping Wu, Ying Cheng, Donghui Chen, Wei Li, Sanyuan Sun, Zhuang Yu, Peiguo Cao, Haihui Chen, Jiejun Wang, Shubin Wang, Hongbing Wang, Songhua Fan, Ye Hua, and Weiguo Su. Effect of fruquintinib vs placebo on overall survival in patients with previously treated metastatic colorec-

tal cancer: The fresco randomized clinical trial. *JAMA*, 319:2486–2496, 6 2018. DOI: 10.1001/jama.2018.7855.

[7] Jin Li, Shukui Qin, Ruihua Xu, Thomas C C Yau, Brigette Ma, Hongming Pan, Jianming Xu, Yuxian Bai, Yihebali Chi, Liwei Wang, Kun-Huei Yeh, Feng Bi, Ying Cheng, Anh Tuan Le, Jen-Kou Lin, Tianshu Liu, Dong Ma, Christian Kappeler, Joachim Kalmus, Tae Won Kim, and CONCUR Investigators. Regorafenib plus best supportive care versus placebo plus best supportive care in asian patients with previously treated metastatic colorectal cancer (concur): a randomised, double-blind, placebo-controlled, phase 3 trial. *The Lancet. Oncology*, 16:619–29, 6 2015. DOI: 10.1016/S1470-2045(15)70156-7.

[8] Per Pfeiffer, Hafdan Sorbye, Camilla Qvortrup, Mia Karlberg, Christian Kersten, Kirsten Vistisen, Birgitta Lindh, Jon Kroll Bjerregaard, and Bengt Glimelius. Maintenance therapy with cetuximab every second week in the first-line treatment of metastatic colorectal cancer: The nordic-7.5 study by the nordic colorectal cancer biomodulation group. *Clinical colorectal cancer*, 14:170–6, 9 2015. DOI: 10.1016/j.clcc.2015.03.002.

[9] Timothy J Price, Marc Peeters, Tae Won Kim, Jin Li, Stefano Cascinu, Paul Ruff, Atilli Satya Suresh, Anne Thomas, Sergei Tjulandin, Kathy Zhang, Swaminathan Murugappan, and Roger Sidhu. Panitumumab versus cetuximab in patients with chemotherapy-refractory wild-type kras exon 2 metastatic colorectal cancer (aspecct): a randomised, multicentre, open-label, non-inferiority phase 3 study. *The Lancet. Oncology*, 15:569–79, 5 2014. DOI: 10.1016/S1470-2045(14)70118-4.

[10] Francesco Sclafani, Tae Y Kim, David Cunningham, Tae W Kim, Josep Tabernero, Hans J Schmoll, Jae K Roh, Sun Y Kim, Young S Park, Tormod K Guren, Eliza Hawkes, Steven J Clarke, David Ferry, Jan-Erik Frödin, Mark Ayers, Michael Nebozhyn, Clare Peckitt, Andrey Loboda, David J Mauro, and David J Watkins. A randomized phase ii/iii study of dalotuzumab in combination with cetuximab and irinotecan in chemorefractory, kras wild-type, metastatic colorectal cancer. *Journal of the National Cancer Institute*, 107:djv258, 12 2015. DOI: 10.1093/jnci/djv258.

[11] Lillian L Siu, Jeremy D Shapiro, Derek J Jonker, Chris S Karapetis, John R Zalcberg, John Simes, Felix Couture, Malcolm J Moore, Timothy J Price, Jehan Siddiqui, Louise M Nott, Danielle Charpentier, Winston Liauw, Michael B Sawyer, Michael Jefford, Nadine M Magoski, Andrew Haydon, Ian Walters, Jolie Ringash, Dongsheng Tu, and Chris J O'Callaghan. Phase iii randomized, placebo-controlled study of cetuximab plus brivanib alaninate versus cetuximab plus placebo in patients with metastatic, chemotherapy-refractory, wild-type k-ras colorectal carcinoma: the ncic clinical trials group and agitg co.20 trial. *Journal of clinical oncology : official journal of the American Society of Clinical Oncology*, 31:2477–84, 7 2013. DOI: 10.1200/JCO.2012.46.0543.

[12] Jianming Xu, Tae Won Kim, Lin Shen, Virote Sriuranpong, Hongming Pan, Ruihua Xu, Weijian Guo, Sae-Won Han, Tianshu Liu, Young Suk Park, Chunmei Shi, Yuxian Bai, Feng Bi, Joong Bae Ahn, Shukui Qin, Qi Li, Changping Wu, Dong Ma, Donghu Lin, and Jin Li. Results of a randomized, double-blind, placebo-controlled, phase

iii trial of trifluridine/tipiracil (tas-102) monotherapy in asian patients with previously treated metastatic colorectal cancer: The terra study. *Journal of clinical oncology : official journal of the American Society of Clinical Oncology*, 36:350–358, 2 2018. DOI: 10.1200/JCO.2017.74.3245.

[13] T Yoshino, J M Cleary, E Van Cutsem, R J Mayer, A Ohtsu, E Shinozaki, A Falcone, K Yamazaki, T Nishina, R Garcia-Carbonero, Y Komatsu, H Baba, G Argilés, A Tsuji, A Sobrero, K Yamaguchi, M Peeters, K Muro, A Zaniboni, N Sugimoto, Y Shimada, Y Tsuji, H S Hochster, T Moriwaki, B Tran, T Esaki, C Hamada, T Tanase, F Benedetti, L Makris, F Yamashita, and H-J Lenz. Neutropenia and survival outcomes in metastatic colorectal cancer patients treated with trifluridine/tipiracil in the recourse and j003 trials. *Annals of oncology : official journal of the European Society for Medical Oncology*, 31:88–95, 1 2020. DOI: 10.1016/j.annonc.2019.10.005.
